# Macro-charcoal accumulation in floodplain wetlands: Problems and prospects for reconstruction of fire regimes and environmental conditions

**DOI:** 10.1371/journal.pone.0224011

**Published:** 2019-10-24

**Authors:** Bradley P. Graves, Timothy J. Ralph, Paul P. Hesse, Kira E. Westaway, Tsuyoshi Kobayashi, Patricia S. Gadd, Debashish Mazumder

**Affiliations:** 1 Department of Environmental Sciences, Faculty of Science and Engineering, Macquarie University, North Ryde, NSW, Australia; 2 Science Division, NSW Office of Environment and Heritage, Sydney South, NSW, Australia; 3 Australian Nuclear Science and Technology Organisation, Lucas Heights, NSW, Australia; University of Pisa, ITALY

## Abstract

Floodplain wetland ecosystems respond dynamically to flooding, fire and geomorphological processes. We employed a combined geomorphological and environmental proxy approach to assess allochthonous and autochthonous macro-charcoal accumulation in the Macquarie Marshes, Australia, with implications for the reconstruction of fire regimes and environmental conditions in large, open-system wetlands. After accounting for fluvial macro-charcoal flux (1.05 ± 0.32 no. cm^-2^ a^-1^), autochthonous macro-charcoal in ~1 m deep sediment profiles spanning ~1.7 ka were highly variable and inconsistent between cores and wetlands (concentrations from 0 to 438 no. cm^-3^, mean accumulation rates from 0 to 3.86 no. cm^-2^ a^-1^). A positive correlation existed between the number of recent fires, satellite-observed ignition points, and macro-charcoal concentrations at the surface of the wetlands. Sedimentology, geochemistry, and carbon stable isotopes (δ^13^C range -15 to -25 ‰) were similar in all cores from both wetlands and varied little with depth. Application of macro-charcoal and other environmental proxy techniques is inherently difficult in large, dynamic wetland systems due to variations in charcoal sources, sediment and charcoal deposition rates, and taphonomic processes. Major problems facing fire history reconstruction using macro-charcoal records in these wetlands include: (1) spatial and temporal variations in fire activity and ash and charcoal products within the wetlands, (2) variations in allochthonous inputs of charcoal from upstream sources, (3) tendency for geomorphic dynamism to affect flow dispersal and sediment and charcoal accumulation, and (4) propensity for post-depositional modification and/or destruction of macro-charcoal by flooding and taphonomic processes. Recognition of complex fire-climate-hydrology-vegetation interactions is essential. High-resolution, multifaceted approaches with reliable geochronologies are required to assess spatial and temporal patterns of fire and to reconstruct in order to interpret wetland fire regimes.

## Introduction

Fire is a significant disturbance agent in the landscape, with impacts on terrestrial flora and fauna, soil and landscape stability, biogeochemical cycles, and human society [1]. Developing palaeofire records allows modern fires to be placed in a long-term context, allowing fire management plans to be developed and adjusted accordingly [2]. Knowledge of past fire regimes can also provide insights into past environmental conditions, such as periods of variability and change in climate (e.g., El Niño Southern Oscillation), hydrology and ecology [3, 4]. For example, climate variations tend to affect vegetation distribution and composition which ultimately alters fire and fuel dynamics across the landscape [5, 6]. Using charcoal and other environmental proxy records to provide information on interactions between fire, hydrology, and vegetation is also critical in sensitive ecosystems [7–9]. To unlock information about long-term fire dynamics, preservation of particulate charcoal must occur in depositional environments, for example, in lakes and wetlands [10]. Human occupation of the landscape and the utilisation of fire as a tool could also potentially influence charcoal assemblages in sedimentary profiles. Furthermore, to place contemporary fire regimes and environmental conditions into a longer-term context and to assess historical anthropogenic impacts on fire, the sediment records also need to extend beyond the influence of recent anthropogenic activity [11].

Macroscopic charcoal (macro-charcoal; sieve size particles >125 μm) has been widely used as a proxy to reconstruct palaeo-fire regimes as it can provide a direct line of evidence of biomass burning in the environment [12–15]. Macro-charcoal preserved in sediment profiles, often expressed as charcoal accumulation rate (CHAR), is considered a reliable indicator of local fire activity [16, 17]. This is based on the assumption that macro-charcoal is the primary charcoal component (peak component), which is rapidly transported and deposited into a waterbody during or shortly after a fire event through fluvial or aeolian (wind) processes [18–20]. In contrast, secondary charcoal (often taking the form of micro-charcoal; sieve size particles <125 μm) is considered to be the background or detrital component introduced to sediment records from afar or during non-fire events by fluvial deposition (e.g., surface run-off), sediment mixing (e.g., bioturbation), or aeolian deposition (e.g., thermal convection or wind) [16–18, 21]. In some cases, however, macro-charcoal can originate from both local and regional (or catchment) sources, suggesting that more complex source-sink processes, depositional conditions, and taphonomic processes may be at work [22].

Wetlands, including lakes, tend to act as sediment sinks by accumulating and storing inorganic matter (e.g., minerals) and organic matter (e.g., charcoal), making them suitable archives for palaeo-environmental research [23]. Most palaeo-fire research has used macro-charcoal in combination with other environmental proxies and were conducted in small, closed-system wetlands with permanently inundated waterbodies situated in high-elevation [8, 24–26] and low-elevation areas [27–30]. This is because these sites are considered to be highly stable and to have high charcoal preservation potential and fairly consistent depositional histories [31]. In open-systems that include rivers or floodplain wetlands, measuring and understanding the relationships between charcoal production, transport, deposition, and preservation is often more challenging [15]. It cannot necessarily be assumed that macro-charcoal is always a direct indicator of local fire activity due to the possibility of external inputs from the riverine corridor and the upstream catchment [18, 32]. Differences in environmental conditions between small, stable closed-systems and large, fluvial and/or dynamic open-systems can further complicate interpretation of fire history in the landscape, and so factors such as catchment size and morphology, stream inputs or outputs, and vegetation types need to be considered [23].

Wetlands in large catchments are important hotspots of sediment accumulation that can record long-term environmental change, but they can also buffer short-term events, creating a lag time between cause and effect within the system, including sediment delivery and water level response [33]. These systems respond to large-scale external catchment controls (e.g., catchment geomorphic processes) which may well respond to changes in climatic conditions and internal factors (e.g., downstream changes in discharge and sediment transport) which affect river behaviour and character [34]. Furthermore, large catchments (e.g., Macquarie catchment; ~26,000 km^2^) with dynamic rivers and wetlands often have complex hydrology and inundation patterns, and fire can be highly variable in space and over time. This means that identifying sources of primary charcoal (i.e., local) and secondary charcoal (i.e., regional) production from within the wetland and elsewhere in the catchment can be problematic. This is particularly true for floodplain wetlands in semi-arid and arid catchments (i.e., wetlands in drylands). These systems have low, variable rainfall, high evapotranspiration, highly variable flooding, and long droughts leading to a mosaic-like geomorphology and vegetation [35, 36]. Although periods of increased fire activity may represent periods with higher fuel loads (i.e., greater biomass), greater ignition potential, or more intense burning [37], it can be difficult to disentangle a fire signal from ‘background noise’ in wetlands in drylands if charcoal comes from multiple sources. Floodplain wetlands with numerous anastomosing and/or distributary channels also have inherently complex geomorphological processes [36]. Understanding the complexity of wetlands, as well as the potential sources of charcoal and processes of charcoal transportation, reworking and deposition are critical for efforts to reconstruct fire activity and interpret fire regimes [10, 17, 23].

The Australian environment has some of the most naturally susceptible and anthropogenic fire-prone landscapes on Earth [38, 39]. The Macquarie Marshes are a large, floodplain wetland system in the Murray-Darling Basin, south-eastern Australia, which regularly burns due to lightning strikes that start fires, graziers burning the reedbeds to promote vegetation regrowth for livestock, and burning activity from indigenous people occupying the landscape [2, 40]. This open-system has multiple tributary inputs and outputs which transport, rework and deposit water, sediment and other organic material from a variety of sources in this hydrologically, geologically and geomorphologically diverse catchment. This setting is quite the opposite of typical small closed-system lake or wetland that have been the focus of most palaeo-fire studies in the past [7, 41, 42]. Closed-system wetlands are often chosen due to their minimal site disturbance, small catchment size, long-term stability and high preservation potential of macro-charcoal found in the sedimentary record. However, long-term fire history or the lasting impacts of fire have rarely been studied in large, open-system floodplain wetlands despite their environmental significance. Nevertheless, management agencies are fully aware of the need for additional information to address pressing environmental issues such as fire in wetlands of high-conservation value, including the Macquarie Marshes [43, 44]. In this paper, we use the Ramsar-listed Macquarie Marshes as an exemplar to apply a combined geomorphological and environmental proxy approach to the analysis of allochthonous (in-situ) and autochthonous macro-charcoal (>125 μm fraction only) accumulation in floodplain wetlands using synthetic grass mats and sediment cores. This study did not assess any micro-charcoal in the <125 μm fraction, charcoal or plant identification, or the role of aeolian processes. We then demonstrate and discuss the problems and prospects for reconstruction of fire regimes and environmental conditions in large, open-system wetlands.

## Study area and field sites

**Fig 1:**
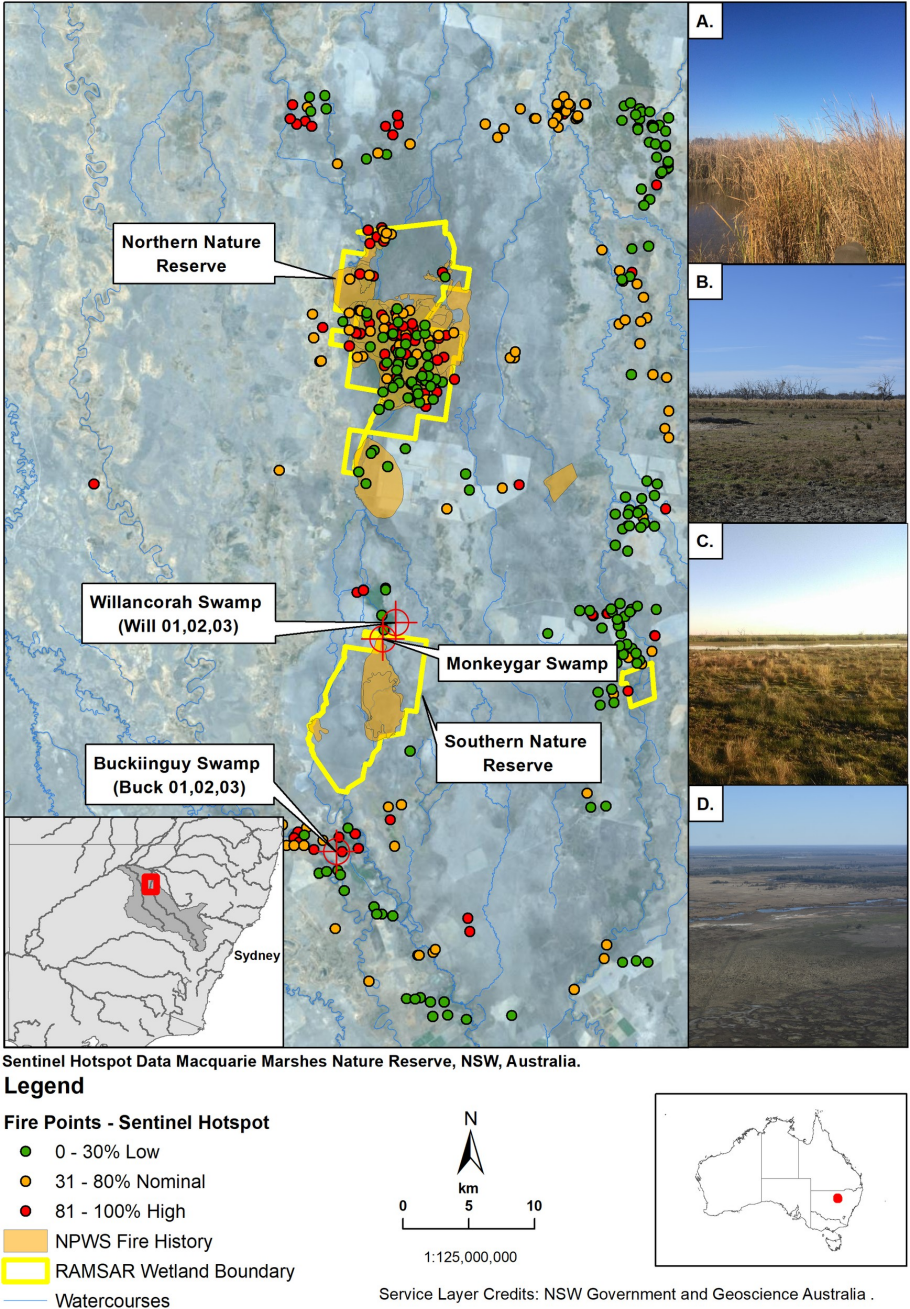
Location of Buckiinguy Swamp and Willancorah Swamp in the Macquarie Marshes, New South Wales, Australia. Ignition point data retrieved from Sentinel Hotspot satellite sensors from 2002–2016 [51], and National Parks and Wildlife Service (NPWS) fire history information [52]. Inset: (A) Reed bed in Buckiinguy Swamp, (B) water couch grassland on the periphery of Buckiinguy Swamp, (C) reed bed (background) and water couch grassland (foreground) near Willancorah Swamp and (D) aerial view of Willancorah Swamp looking northwest. Service layer credits: NSW Government [53], and Geoscience Australia [54].

The Macquarie River is located in central New South Wales and drains a catchment area of ~26,000 km^2^ [35]. The Macquarie Marshes (30° 45’ S, 147° 33’ E), located on the alluvial plain fed by this catchment, has a diverse network of river channels and permanent, intermittent and ephemeral wetlands located on the lower reaches of the Macquarie River, New South Wales (Fig 1). The wetland system covers an area of ~ 2,500 km^2^ when inundated during large floods, and is situated in a semi-arid climate zone (Köppen classification BSh) with mean annual rainfall ~442 mm and mean annual evapotranspiration >2,000 mm (Quambone rainfall gauge 051042) [45]. The aeolian environment is subject to zonal westerly winds in winter, easterly trade winds, and convective thunderstorms in summer.

The geomorphological record of the Macquarie Marshes reveals a long history of channel formation and abandonment due to avulsion (the process of channel relocation on the floodplain) driven by sedimentation and erosion, as well as wetland adjustment resulting from changes in inundation and ecosystem processes [46]. The more regularly inundated wetlands have extensive areas of common reed (*Phragmites australis*), bulrush (*Typha domingensis*), water couch (*Paspalum distichum*) grassland, and river cooba (*Acacia stenophylla*) woodland and river red gum (*Eucalyptus camaldulensis*) woodland/forest that are reliant on overbank and overland flooding from numerous channels [35, 47]. The irregular channel and floodplain morphology in the southern Macquarie Marshes is characterised almost entirely by muds (Vertisol soil type) [46–48]. The wetlands provide essential habitat and refuge for a wide range of flora and fauna [44, 49]. The Macquarie Marshes Nature Reserve is concentrated on two core areas in the southern and northern parts of the system, which comprise ~10% (~219 km^2^) of the total wetland area. The nature reserve is recognised under the Ramsar Convention for its international ecological significance, namely a prime location for migratory, colonial and endangered waterbirds [44, 49, 50].

The Macquarie Marshes have been the focus of much research on hydrology [55–57], ecology [49, 58–61], geomorphology [35, 47, 62], and environmental management [43, 63] over the past two decades. However, very little is known about the fire history in this system despite the propensity for the wetlands to be ignited by lightning strikes, the probability of a long history of Aboriginal burning [40], and the fact that fire has been used by pastoralists in the European settlement period for the management of wetland vegetation and to promote regrowth for cattle and sheep grazing [2]. Of the 18 major wildfires since 1947 in and adjacent to the nature reserve, four of the largest fires (1947, 1966, 1994 and 1995) burnt >10 km^2^ of reed beds, grassland and riverine woodlands in the northern Macquarie Marshes [2, 44]. These events were perceived as being rare, because seasonal flooding did not occur in these years due to the onset of drought conditions [2]. However prior to the 1960s and before the effects of major river regulation (e.g., Burrendong Dam, built 1967), fires occurred every one or two years but they were typically short-lived and burnt out within a few days partly due to the sub-surface moisture protecting the roots and the reedbeds [44]. Regardless of the ignition process or severity of burning, significant fire events in reedbeds and woodlands should leave a macro-charcoal record in sediment, which could be used to reconstruct fire history in the wetlands.

Buckiinguy Swamp and Willancorah Swamp receive regular (typically annual) inundation and are key ecological assets under private ownership adjacent to the southern nature reserve (Fig 1) that support a variety of semi-permanent and flood-dependant vegetation, dominated by reeds and rushes [50, 64]. For a variety of reasons both wetlands are susceptible to burning by fire. Buckiinguy Swamp is a floodout-style wetland at the end of Buckiinguy Creek, which is a distributary channel of the Macquarie River. The wetland developed on top of sediment laid down by the Macquarie River and is mainly occupied by a small reed bed with water couch grassland, and river red gum woodland at its margins (Fig 1A and 1B). Buckiinguy Creek breaks down and terminates as it flows into the reed bed due to overbank water loss, sedimentation in the channel, and in the wetland, and small channels on the northern perimeter drain water back into the Macquarie River [47]. Willancorah Swamp is also a floodout-style wetland with a large reed bed, water couch grassland and some river red gum woodland (Fig 1C and 1D). This wetland is fed by Monkeygar Creek, a distributary channel of the Macquarie River until the early 20^th^ century when it became the main course of the river in the southern Macquarie Marshes [46, 47]. Monkeygar Creek breaks down into Willancorah Swamp for the same reasons as Buckiinguy–a loss of flow and sediment transport efficiency–and has several channels exiting at the north and back into the Macquarie River.

## Methods

Fieldwork was conducted on private property and no further access to any of the northern or southern Macquarie Marshes was needed. Access to the private property where field sites were located (for this study) was given from Mr David and John Thornton (Buckiinguy Managers) and Willancorah Swamp was Mr Matt Bell (Willancorah Manager). In addition, the research activities that took place had no involvement with any endangered or protected species. All relevant principle data is in the supporting information folder, and available on request from the corresponding author.

### Ignition hotspot mapping

Mapping and spatial analysis of ignition points in the Macquarie Marshes from 2002–2016 was performed using the Geoscience Australia Sentinel Hotspot database, part of the national bushfire monitoring system of Australia. Sensors on satellites measure the radiative power (MW km^-2^) of ignition points which are then assigned a confidence level where: 0–30% is ‘Low’; 30–80% is ‘Nominal’ and >80% is ‘High’ confidence [65, 66]. These ignition points are sites of likely fire activity (Fig 1). Complementing the Sentinel ignition data is wildfire and prescribed burn data of known fire events from the New South Wales National Parks and Wildlife Service (NPWS) which have also been included in the mapping and spatial analysis (Fig 1) [52].

### Fluvial sediment and charcoal sampling

In a previous study [67], synthetic grass mats (surface area 0.4 m^2^) were anchored to the ground in the wetlands adjacent to Buckiinguy Creek and in Buckiinguy Swamp prior to an environmental water release and a series of small floods which caused inundation for 9 months in 2009–2010. No fires occurred within the sampling area or swamp during this period of sediment sampling and so any charcoal in the sediment is of allochthonous origin. The mats trapped contemporary fluvial sediment washed into the wetlands ~2 km upstream of the reed bed (site B1); 0.25 km upstream of the reed bed (site B2); at the entry to the reed bed (site B3), and; at the downstream edge of the reed bed (site B4). Each site had three sampling intervals at increasing distances away from the channel (e.g., 2 m, 10 m, 50 m) and at each interval, mats were laid in a 2 x 2 m grid (i.e., 4 mats per interval, 3 intervals per site, 4 sites; n = 48). The mats from all sites along Buckiinguy Creek and in the wetland were used to calculate the allochthonous charcoal and sediment load. After 9 months of inundation, the mats were collected and the fluvial sediment and macro-charcoal deposited and trapped on the mats were extracted, weighed and dried for analysis [67]. After calculating macro-charcoal concentration (i.e., no. cm^3^) and macro-charcoal flux (i.e. no. cm^-2^ a^-1^) for each synthetic-grass mat at each site (B1-B4), an average flux value was derived for the allochthonous contribution, or fluvial background, representing macro-charcoal from any origin entering the wetland from upstream. The allochthonous contribution was then applied to the total charcoal accumulation rate (i.e., CHAR; no. cm^-2^ a^-1^) from each sediment core to isolate the autochthonous, or *in situ*, macro-charcoal signal potentially related to local fires in the wetlands.

### Core sampling and sedimentology

Three sediment cores were collected from each wetland. Buck01 (53 cm) and Buck02 (54 cm) from Buckiinguy, and Will01 (50 cm) and Will02 (60 cm), from Willancorah, were collected in November 2016. In April 2017, Will03 (117 cm) was collected. Buck03 (85 cm) was collected in 2001 by Ralph (2001) and stored sealed in a freezer until it was opened in 2016. The coring method consisted of using a PVC pipe enclosed in a steel push tube with a steel cutting shoe and impact protection plug designed for these dense, usually dry, clay-rich sediments. Once the steel core was assembled it was driven into undisturbed sediment using a slide hammer (i.e., star picket hammer). The core was carefully extracted manually by using two high-lift jacks to ensure no loss of sample from the cutting shoe.

Coring was restricted to the margins of the wetlands due to inundation and the inoperability of the coring setup in deep standing water, but the sampling sites were representative of the wetlands and had minimal disturbance. Buck01 and Buck02 were situated next to each other in a water couch grass meadow. Buck03 was taken from the centre of the Buckiinguy reed bed [68]. Will01 and Will02 were situated next to each other in a water couch grass meadow, while Will03 was on the edge of the reed bed ~17 m west of Will02. Buck01 and Will01 were used for geochronology. Buck02, Buck03, Will02 and Will03 were used for sedimentology, charcoal and stable isotope analyses, and half of each core was kept intact for geochemistry analysis using micro X-ray fluorescence (XRF) core scanning (Itrax).

Water content and dry bulk density (DBD) were determined using standard methods [69], before samples were wet-sieved at 63 μm to determine the >63 μm (sand) and <63 μm (mud; silt plus clay) fractions. Loss-on-ignition (LOI) was performed at 550°C (LOI_550_) to determine organic matter content, and at 950°C (LOI_950_) to determine inorganic matter (carbonate) content [70].

### Charcoal extraction and analysis

Macro-charcoal extraction, dispersal and counting procedure followed Stevenson and Haberle [71]. Sub-samples of ~2.26 cm^-3^ were taken continuously at 1.5 cm intervals along the length of each core and the >250 μm and 125–250 μm macro-charcoal fractions were isolated. Together these represent total macro-charcoal concentration in each sub-sample (>125 μm; no. cm^-3^). Charcoal accumulation rates (CHAR; no. cm^-2^ a^-1^) were calculated by multiplying the macro-charcoal concentration of each sub-sample by the mean linear sedimentation rate derived from optically-stimulated luminescence (OSL) dating (see geochronology, below). Mean CHAR was then determined for all sub-samples in each core. The depth axis for each core was converted to age by dividing each depth increment by the mean linear sedimentation rate.

### Stable isotope analysis

Physical and chemical treatment for carbon stable isotope (δ^13^C) and total carbon (C %) analyses was done at the Australian Nuclear Science and Technology Organisation (ANSTO) using standard methods [72, 73]. Samples were prepared with 1M HCl to remove carbonates prior to δ^13^C analysis, and then analysed using an Elementar VarioMICRO Elemental Analyser and an IsoPrime Continuous-Flow Isotope Ratio Mass Spectrometer (CF-IRMS) to quantify the variations of carbon isotopes. The results are reported as δ^13^C values in parts per thousand (‰ or ‘per mil’), where δ^13^C refers to the ratio of ^13^C:^12^C relative to an internationally defined scale Vienna Pee Dee Belemnite standards (VPDB). Total carbon analysis was performed separately on an untreated sub-sample using the same machine as mentioned above.

### Sediment geochemistry

Sediment geochemistry was analysed using Itrax high-resolution core scanning and micro-X-ray fluorescence (micro-XRF) spectrometry at ANSTO. The Cox Analytical System Itrax system provides high-resolution optical images, X-radiographs, geochemical and magnetic susceptibility profiles. The technique is non-destructive and provides data as counts which are considered semi-quantitative values for elemental composition [74]. The resolution for the X-radiograph was 500 μm and the XRF analysis was 1000 μm. Magnetic susceptibility was measured at 5 mm intervals. All results were processed using Q-Spec software and normalised by the total counts to correct for porosity and matrix [75]. Principal component analysis (PCA) was carried out using RStudio.

### Geochronology

A chronology for the sediment cores from Buckiinguy and Willancorah Swamp was established using optically-stimulated luminescence (OSL) dating. Cores were split longitudinally and sub-sampled under subdued red-light conditions. The sub-samples were processed according to the techniques of Wintle and Murray [76] to isolate the 90–212 μm fraction of quartz, including a 40% hydrofluoric acid etch for 45 minutes to remove the alpha irradiated outer surface of the quartz grains [77].

Prior to single-grain analysis, preheat plateau and dose recovery tests were conducted to determine the optimal measurement conditions. From these tests a preheat temperature of 260°C for 10 seconds was chosen, which could recover a surrogate dose of 100 Gy. All OSL measurements were conducted in an automated Risø TL-DA-20 reader fitted with an Electron Tubes Ltd 9635QA photomultiplier tube and 3 x U340 filters. For single-grain OSL measurements, quartz grains from the 180–212 μm fraction were mounted on a 10x10 precision drilled aluminium disk. The grains were stimulated for 2 seconds using a green 10 Mw 532 nm Nd:YVO4 solid-state diode pump laser. The single-aliquot regeneration (SAR) protocol was applied for single-grain analysis for equivalent dose determination [78, 79]. Acceptance or rejection of grains was based from the criteria outlined in Jacobs *et al*. [80].

The contribution of Uranium, Thorium, and Potassium to the dosimetry of the surrounding sediment was estimated using a Geiger-Muller beta counter for dried and milled sediment combined with thick source alpha counting using a Daybreak 583 alpha counter. Cosmic ray contribution including altitude, geomagnetic latitude, sediment overburden, and water content during sediment burial was also included [81]. The equivalent dose was divided by the environmental dose rate to derive an OSL age estimate, which was used to create age-depth model where the y-intercept was set to zero age at the surface in order to calculate linear sedimentation rates [77, 82]. The statistical model chosen for the burial age was based on the fluvial environmental setting and dispersion of single grain equivalent dose [78, 83].

## Results

### Ignition hotspots and fire history

**Fig 2:**
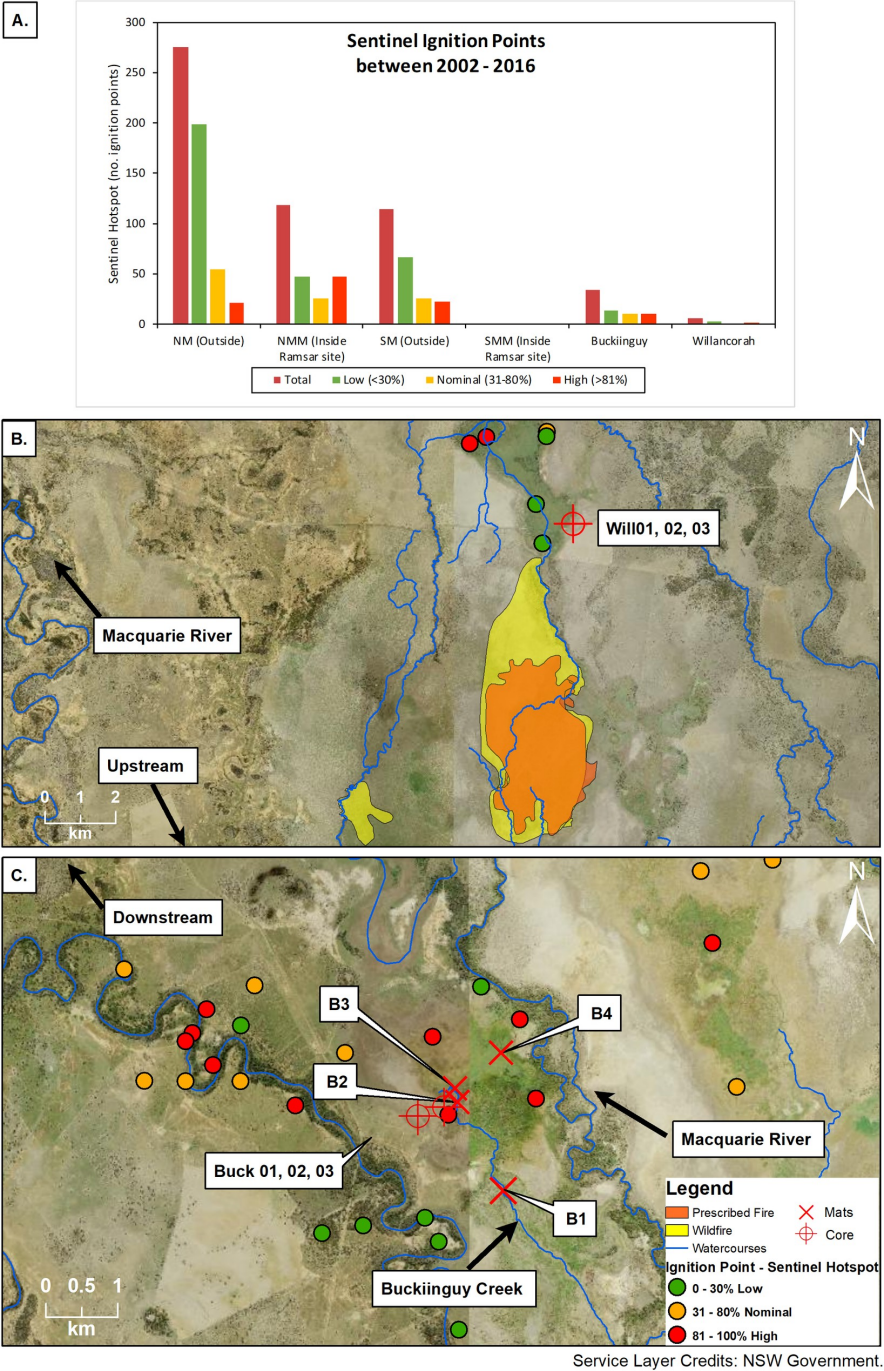
Fire ignition point data from Sentinel Hotspot. As shown: (A) summary of low, nominal and high confidence information from 2002–2016 for the northern and southern Macquarie Marshes, (B) distribution of fire ignition points in Willancorah Swamp 2002–2016, and (C) distribution of fire ignition points in Buckiinguy Swamp 2002–2016. Wildfire and prescribed burn information show the culmination of maximum burn area between 1980–2004 in Monkeygar Swamp (upstream from Willancorah Swamp). Information retrieved from Geoscience Australia [51], NPWS Fire History [52], and satellite imagery figures provided by NSW Government [53].

Sentinel Hotspot information reveal several clusters of ignition points in the northern and southern Macquarie Marshes (Fig 1). The northern Macquarie Marshes have had by far the greatest number of ignition points in the last 10 years, many of these being inside the nature reserve (Fig 2). NPWS fire information also shows there were 16 recorded wildfires in the northern Marshes from 1978–2016, and 15 prescribed burns from 1987–2016 [52]. In contrast, the Sentinel Hotspot information shows that the southern nature reserve had no ignition points in the last 10 years, however, these have occurred adjacent to the southern nature reserve in Buckiinguy and Willancorah Swamp. Buckiinguy experienced 33 ignition points in the period 2002–2016 (Fig 2), while Willancorah Swamp had just six (Fig 2). At Buckiinguy, several of the high confidence ignition point hotspots were in close proximity to the sediment coring sites, although NPWS recorded no wildfire or prescribed burns [52]. At Willancorah there are no ignition point hotspots close to the coring sites and NPWS information shows no wildfire or prescribed burns in this area either. However, in the southern nature reserve, Monkeygar Creek (upstream from Willancorah Swamp) has experienced four wildfires from 1980–2004 and eight prescribed burns from 1992–2004 [52]. NSW government records show that since 1947 there were 18 known major wildfires in and adjacent to wetlands in what is now the Macquarie Marshes Nature Reserve [44]. Currently, there is no longer-term record of fire activity to place these recent fire events in context.

### Allochthonous macro-charcoal accumulation on mats

**Fig 3:**
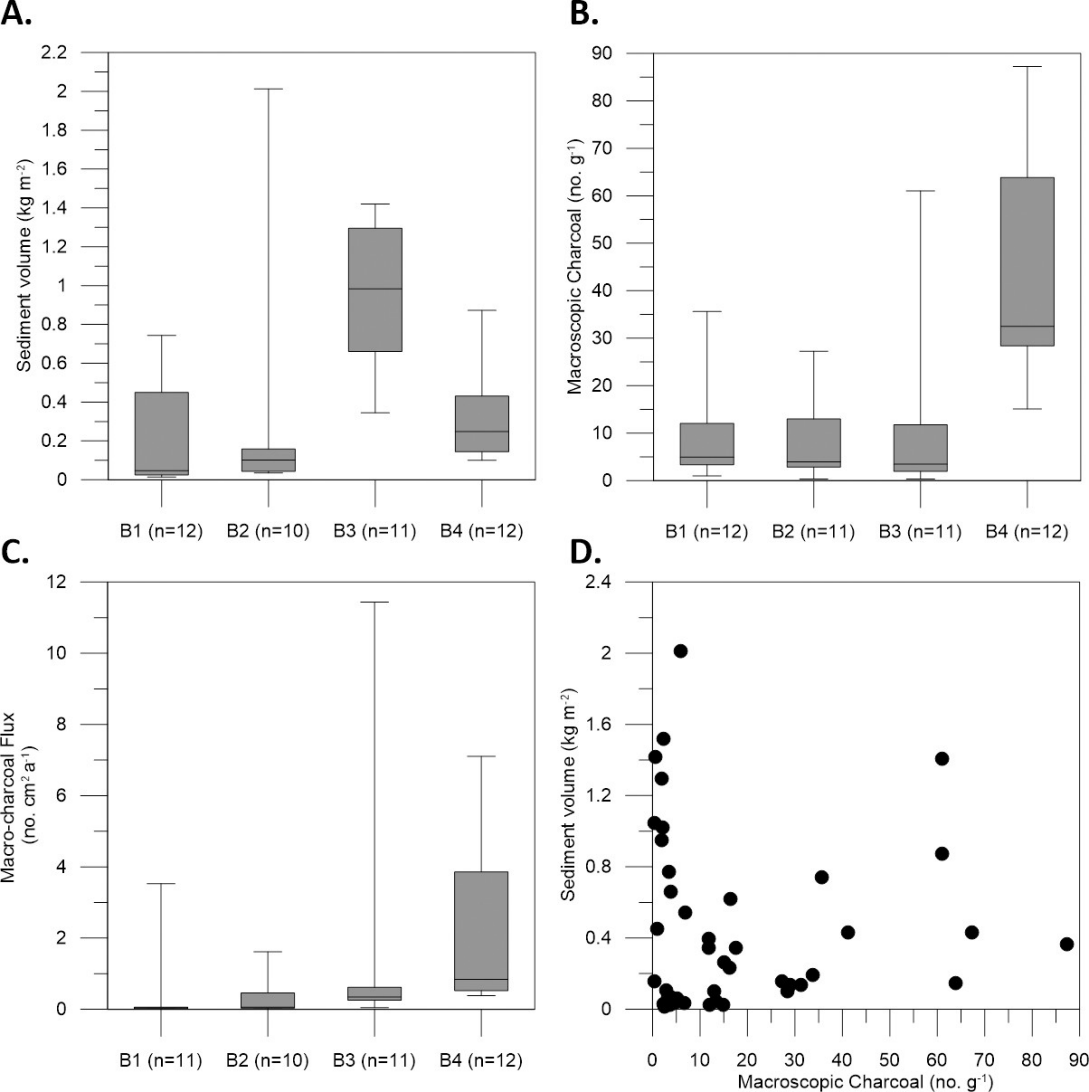
Fluvial sediment and macro-charcoal in Buckiinguy Swamp. Demonstrates the: (A) volume of deposited sediment at sites B1, B2, B3, and B4, (B) macro-charcoal concentration at sites B1, B2, B3 and B4, (C) allochthonous macro-charcoal flux, and (D) the relationship between fluvially deposited sediment and macro-charcoal concentration after 9 months of inundation in 2009–2010.

The distribution of fine sediment trapped on synthetic grass mats in Buckiinguy Swamp during 9 months of inundation in 2009–2010 was highly variable, but some clear trends are present (Fig 3). Site B3 where the channel of Buckiinguy Creek enters the reed bed had the highest volume of deposited sediment, while sites B1 and B2 upstream and site B4 downstream had variable, but lower volumes of deposited sediment (Fig 3A). All three sites along Buckiinguy Creek leading into the reed bed (B1, B2, and B3) revealed consistently low concentrations of macro-charcoal in the modern deposited sediment, demonstrating fluvial sediment derived from the upstream catchment has a generally low concentration of macro-charcoal during this sampling interval (Fig 3B). Site B4 in the Buckiinguy Swamp reed bed had slightly higher and more variable concentrations of macro-charcoal in sediment deposits. Macro-charcoal flux was low at sites B1, B2, and B3, with a slight increase in the mean value at site B4 (Fig 3C). The mean (and standard error) of the baseline of fluvial charcoal entering the system from upstream was determined to be 1.05 ± 0.32 no. cm^-2^ a^-1^ based on data from all sites (Fig 3C). There was no significant linear relationship (r^2^ = 0.0002; p = 0.95) between the amount of fluvially deposited sediment and macro-charcoal concentration at all the sites in Buckiinguy Swamp (Fig 3D).

**Fig 4:**
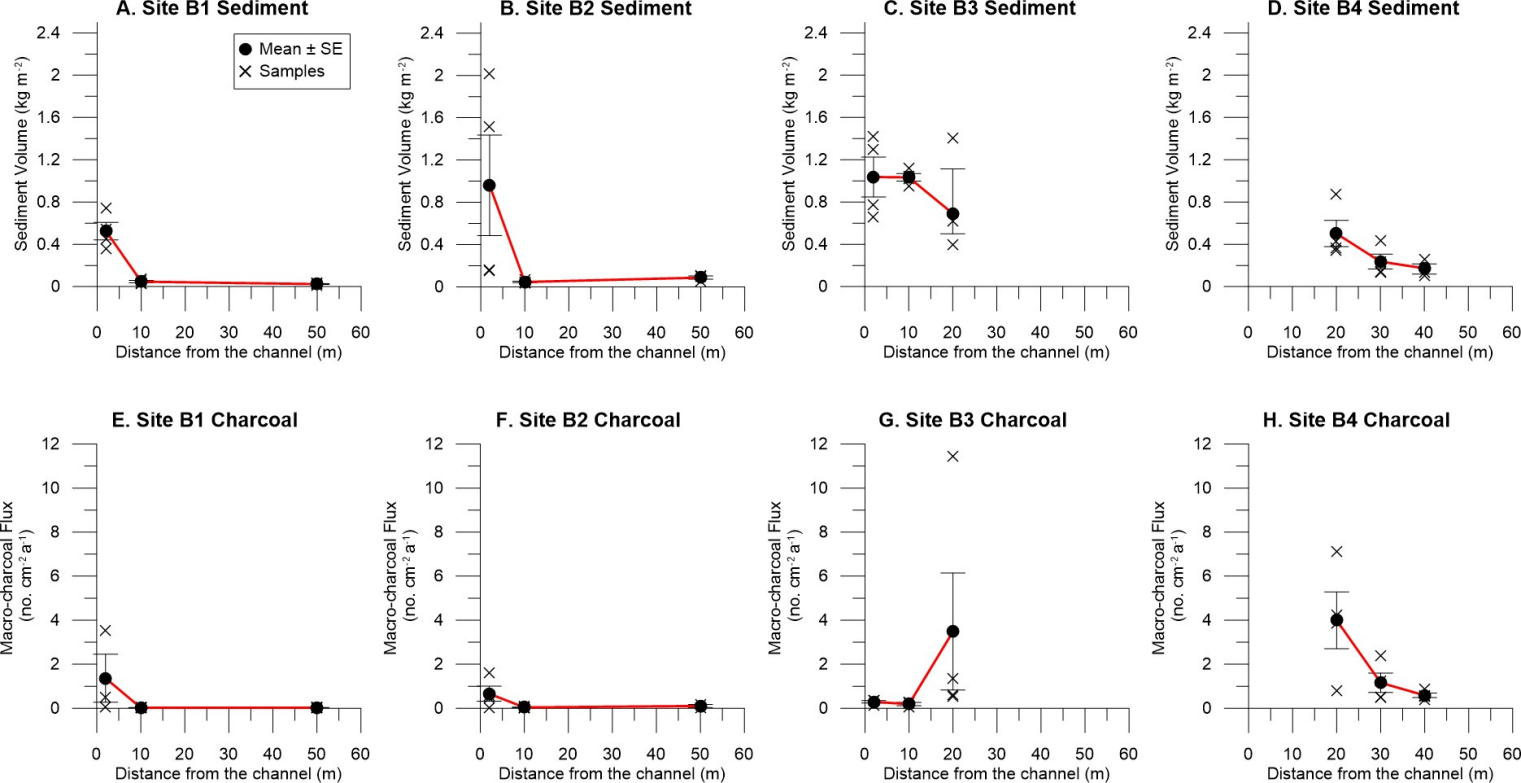
Volume of sediment deposited with increasing distance from the channel in Buckiinguy Swamp. As shown above: (A) site B1, (B) site B2, (C) site B3, and (D) site B4, and charcoal flux in the deposited sediment in Buckiinguy Swamp at (E) site B1, (F) site B2, (G) site B3, and (H) site B4.

Variability and lateral trends were observed in deposited sediment and macro-charcoal at each of the sites. Samples closest to the channel of Buckiinguy Creek had more variable and greater volumes of deposited sediment than sites further away from the channel (Fig 4A–4D). Downstream along Buckiinguy Creek, there are some trends with macro-charcoal accumulation with distance away from the channel. Sites B1 and B2 seem to decline slightly in macro-charcoal onto the floodplain, while site B3 an increase in macro-charcoal across the floodplain, and site B4 has a decrease in macro-charcoal with distance (Fig 4E–4H).

The average fluvial macro-charcoal flux across all sites representing a ‘baseline’ allochthonous value (1.05 ± 0.32 no. cm^-2^ a^-1^) can be subtracted from the total macro-charcoal accumulation rates found in sediment cores from the wetlands, to yield an *in situ* macro-charcoal record most likely related to local fires in the wetlands. When the fluvial macro-charcoal background is considered in the sediment cores, small peaks below the background are removed, leaving only the larger peaks in the record.

### Core sedimentology and autochthonous macro-charcoal

**Fig 5:**
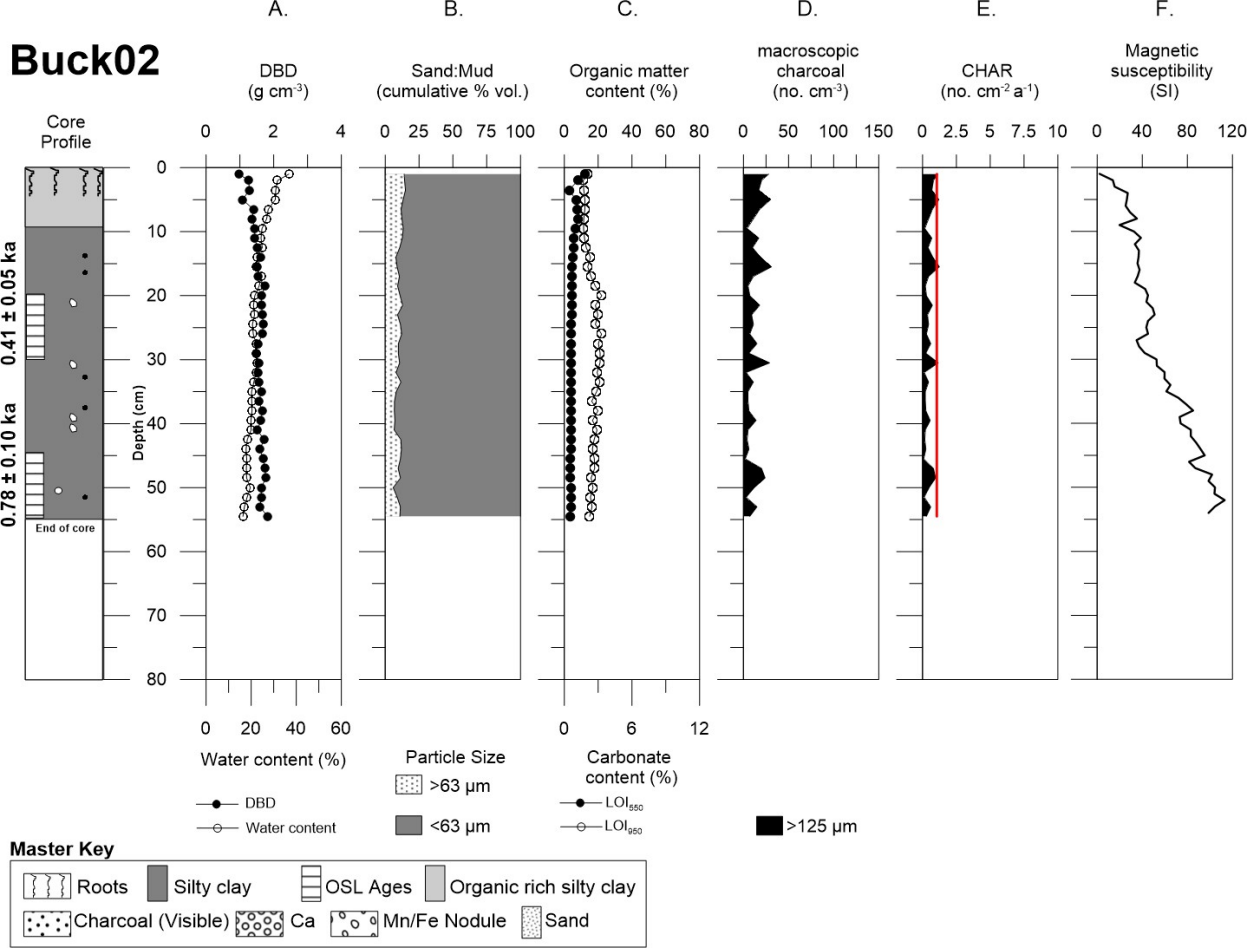
Detailed core description of Buck02. Demonstrates Buck02 with: (A) DBD and water content, (B) sand and mud content, (C) LOI_550_ and LOI_950_, (D) autochthonous macro-charcoal concentration, (E) charcoal accumulation rate (redline represents the allochthonous background flux), and (F) magnetic susceptibility.

**Fig 6:**
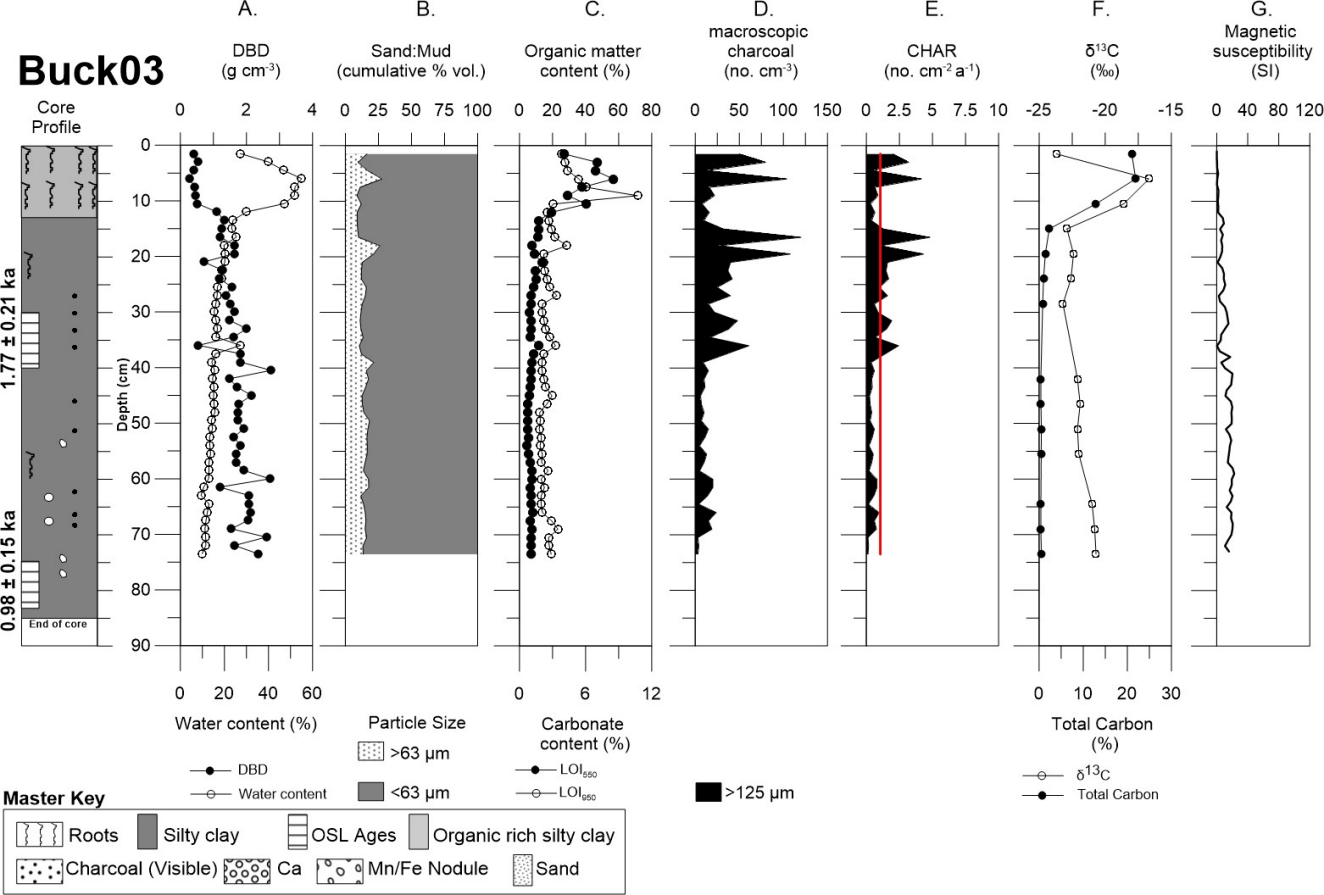
Detailed core description of Buck03. Demonstrates Buck03 with: (A) DBD and water content, (B) sand and mud content, (C) LOI550 and LOI950, (D) autochthonous macro-charcoal concentration, (E) charcoal accumulation rate (redline represents the allochthonous background flux), (F) δ13C and total carbon, and (G) magnetic susceptibility.

Buckiinguy Swamp cores Buck02 and Buck03 consisted of relatively organic rich mud (i.e., silt plus clay fraction) with some roots present above ~10 cm of the cores (Figs 5 and 6). Buck02 sand content (>63 μm) above 12.5 cm was (~13%), followed with a relatively minor decline in sand content (~9.6%) with increasing depth, whereas mud dominated (~89%) of the core. Organic matter and carbonate content were quite uniform with depth in Buck02 (Fig 5). Sand content for Buck03 revealed two minor peaks at the depth of 6 cm (27.6%) and 18 cm (26%) with no significant changes in the remaining sand content (~14%) below 18 cm, the remaining mud fraction was dominant (~85%) for the entire core (Fig 6). Visible roots, charcoal fragments, manganese/iron nodules, and carbonate nodules occurred throughout the profiles. DBD was lowest near the surface and increased with depth (from 0.2 to ~2.0 g cm^-3^). Organic matter (LOI_550_) and carbonate (LOI_950_) content were highest in the upper ~10 cm of the profile for Buck03 (~54% and 12%, respectively) before decreasing with depth (Fig 6).

Macro-charcoal concentrations (i.e., raw counts) are variable in both cores from Buckiinguy. Buck02 has intermittent gaps with small macro-charcoal peaks at depths of ~5 cm, ~15 cm, ~30 cm and ~48 cm (Fig 5). In contrast, Buck03 has consistently higher macro-charcoal concentrations throughout the upper 40 cm of the profile, with distinctive peaks at depths of ~4 cm, ~8 cm and ~20 cm, as well as a series of smaller maximum concentrations in between (Fig 6). Furthermore, there is a small increase in coarser sediment (>63 μm) at a depth of 18 cm that coincides in-between two macro-charcoal peaks at 16.5–19.8 cm (See Fig 6B). Both cores from Buckiinguy had relatively high macro-charcoal concentrations at the surface.

**Fig 7:**
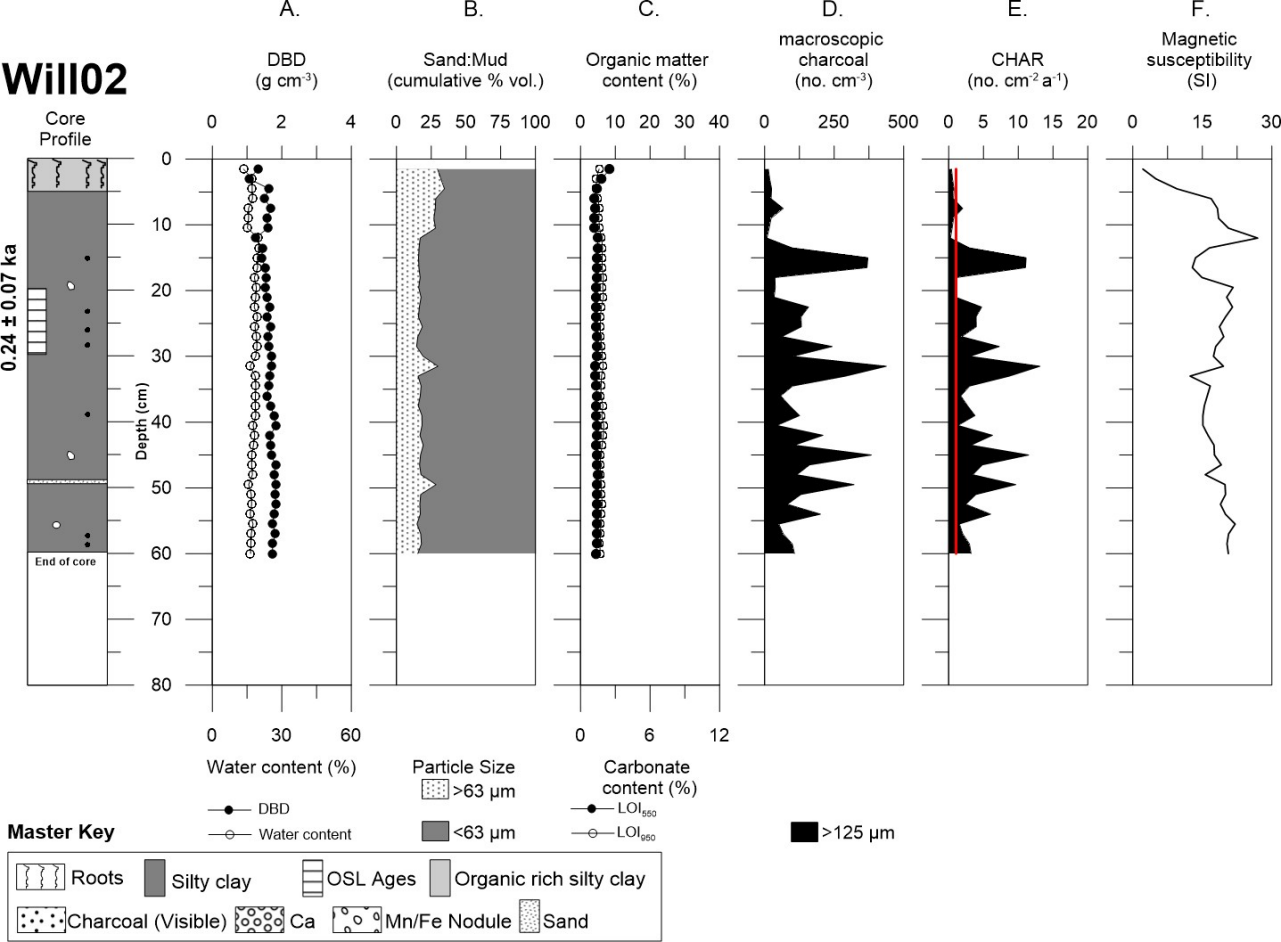
Detailed core description of Will02. Demonstrates Will02 with: (A) DBD and water content, (B) sand and mud content, (C) LOI_550_ and LOI_950_, (D) autochthonous macro-charcoal concentration, (E) charcoal accumulation rate (redline represents the allochthonous background flux), and (F) magnetic susceptibility.

**Fig 8:**
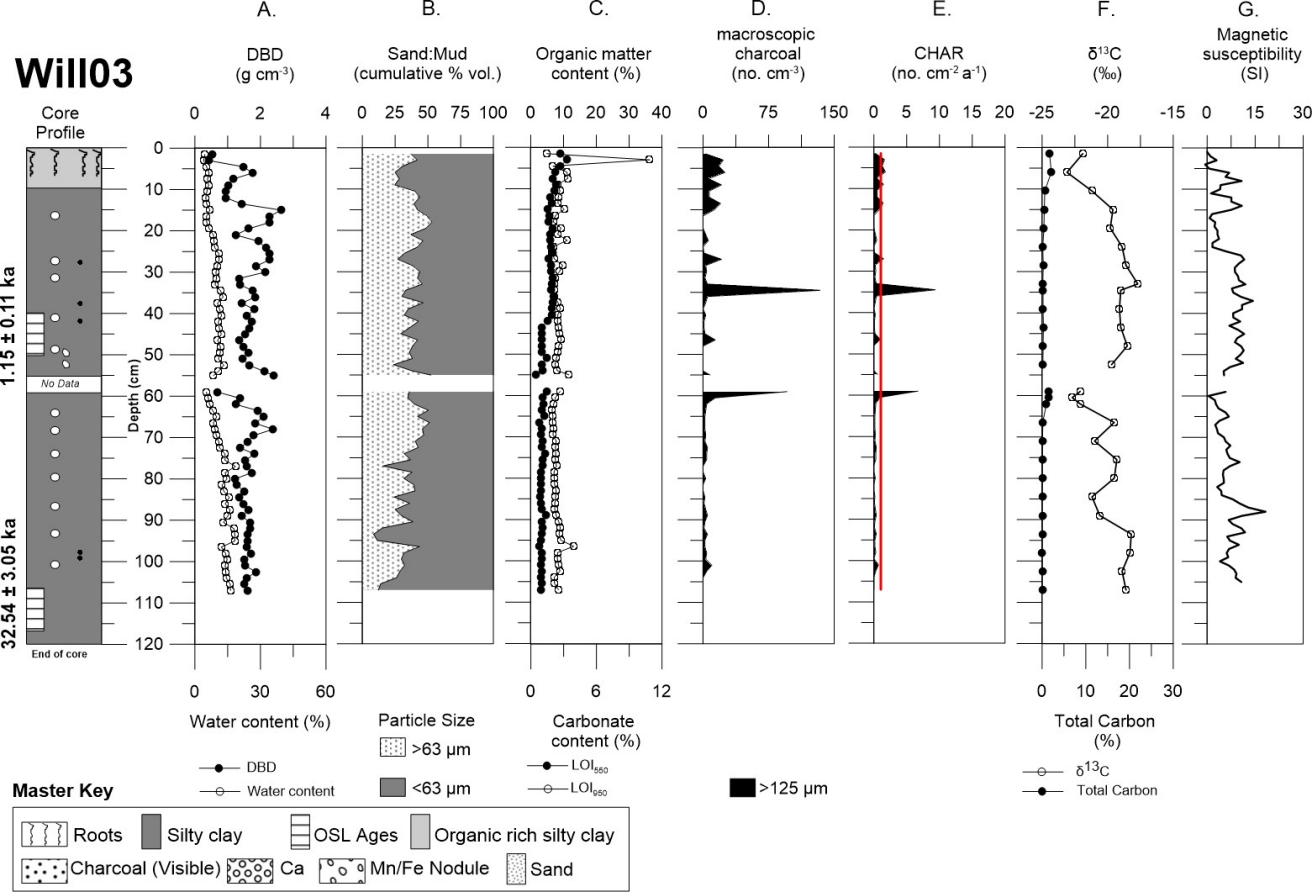
Detailed core description of Will03. Demonstrates Will03 with: (A) DBD and water content, (B) sand and mud content, (C) LOI_550_ and LOI_950_, (D) autochthonous macro-charcoal concentration, (E) charcoal accumulation rate (redline represents the allochthonous background flux), (F) δ^13^C and total carbon, and (G) magnetic susceptibility.

Willancorah swamp cores Will02 and Will03 also consisted of organic rich, silty clay in the upper 5–10 cm with some roots towards the surface of the profile (Figs 7 and 8). Will02 sand content above 10.5 cm was (~29%) followed by a small decrease in sand (~17%) with depth, excluding the two notable peaks at 31.5 cm (29.8%) and 49.5 cm (28.5%), although the mud fraction (~82%) was still dominant throughout the core (Fig 7). However, Will03 sand content was noticeably different with respect to the other cores, it is higher and variable (~35%), although the mud content remained the dominant fraction (~65%) throughout the core. Additionally, there was an abundance of calcium carbonate nodules throughout the profile, which was complemented with flecks of charcoal, manganese, and iron nodules below a depth of ~25 cm (Fig 8). DBD was also similar throughout the cores (1.5 to 2.0 g cm^-3^), except for a peak in carbonate at ~3 cm in Will03. Both organic matter and carbonate content were uniform with depth in the cores.

Macro-charcoal concentrations for Will02 and Will03 were highly variable with depth. Will02 had very high concentrations of macro-charcoal and several peaks throughout the profile between 8–60 cm (Fig 7D). In contrast, Will03 had much lower concentrations of macro-charcoal and just two peaks at ~37 cm and ~59 cm (Fig 8D). Both cores from Willancorah had relatively low macro-charcoal concentrations at the surface.

### OSL dating and Charcoal Accumulation Rate (CHAR)

The single-grain (SG) OSL age of 0.41 ± 0.05 ka for 20–30 cm sediment in Buck02, is stratigraphically consistent with the 0.78 ± 0.10 ka age for sediment at a depth of 42–52 cm (Table 1, S1 Fig). The OSL ages determined for Buck03 were 1.77 ± 0.21 ka at 30–40 cm and 0.98 ± 0.15 ka at 75–85 cm. However, the Buck03 sample at 75–85 cm only produced a small number of quartz grains, and thus, its resulting age estimate was not included in the age-depth model for this core.

**Table 1:** Summary details of single-grain OSL ages for Buck02, Buck03, Will02 and Will03, showing dose rate, equivalent dose, and minimum-age-model ages.

Sample	Depth	Grain	Beta	Gamma	Cosmic-ray	Water	Total	Dispersion	Accepted	Equivalent	Age ^i^
code		Size	dose rate^a^	dose rate^b^	dose rate^c^	content^d^	dose rate^e^		grains^f^	dose^g^^,^ ^h^	
* *	(cm)	(μm)	(Gy ka^-1^)	(Gy ka^-1^)	(Gy ka^-1^)	(%)	(Gy ka^-1^)	(%)	(%)	(Gy)	(ka)
**Buck02**	20–30	90–212	1.252 ± 0.056	0.678 ± 0.392	0.155 ± 0.015	25 ± 5	2.439 ± 0.152	129.6	22.2	1.00 ± 0.10	**0.41 ± 0.05**
**Buck02**	42–52	90–212	1.252 ± 0.056	0.678 ± 0.392	0.152 ± 0.015	25 ± 5	2.437 ± 0.152	95.7	25.5	1.90 ± 0.20	**0.78 ± 0.10**
**Buck03**	30–40	90–212	0.386 ± 0.143	0.511 ± 0.004	0.163 ± 0.016	18 ± 5	1.188 ± 0.114	117.5	37.8	2.10 ± 0.11	**1.77 ± 0.21**
**Buck03**	75–85	90–212	0.386 ± 0.143	0.511 ± 0.004	0.158 ± 0.016	18 ± 5	1.183 ± 0.114	186.5	14.0	1.16 ± 0.12	**0.98 ± 0.15**
**Will02**	20–30	90–212	0.966 ± 0.037	0.657 ± 0.390	0.160 ± 0.016	20 ± 5	2.067 ± 0.135	133.5	15.6	0.85 ± 0.13	**0.24 ± 0.07**
**Will03**	40–50	90–212	0.494 ± 0.031	1.117 ± 0.645	0.144 ± 0.015	30 ± 5	1.819 ± 0.141	131.7	17.0	2.10 ± 0.10	**1.15 ± 0.11**
**Will03**	107–117	90–212	0.543 ± 0.031	1.117 ± 0.645	0.152 ± 0.016	20 ± 5	1.875 ± 0.147	40.2	9.0	61.00 ± 2.40	**32.54 ± 3.05**

a. Concentrations determined from Geiger-Muller beta counter measurements of dried and powdered sediment samples.

b. U and Th from Daybreak 583 thick source alpha counter and combined with Geiger-Muller beta counter to calculate K.

c. Time-averaged cosmic-ray dose rates (for dry samples), each assigned an uncertainty of ± 10%.

d. Field / time-averaged water contents, expressed as (mass of water/mass of dry sample) x 100. The latter values were used to calculate the total dose rates and OSL/TL ages.

e. Mean ± total (1σ) uncertainty, calculated as the quadratic sum of the random and systematic uncertainties. An internal dose rate of 0.03 Gy ka-1 is also included.

f. Number of grains processed/number of accepted grains for the samples. Low numbers of processes and accepted grains were due to the small amount of quartz yielded for certain samples.

g. Minimum age model [78] used to determine the equivalent dose due to the high over-dispersion and depositional environment.

h. Palaeodoses include a ± 2% systematic uncertainty associated with laboratory beta-source calibrations.

i. Uncertainties at 68% confidence interval.

**Fig 9:**
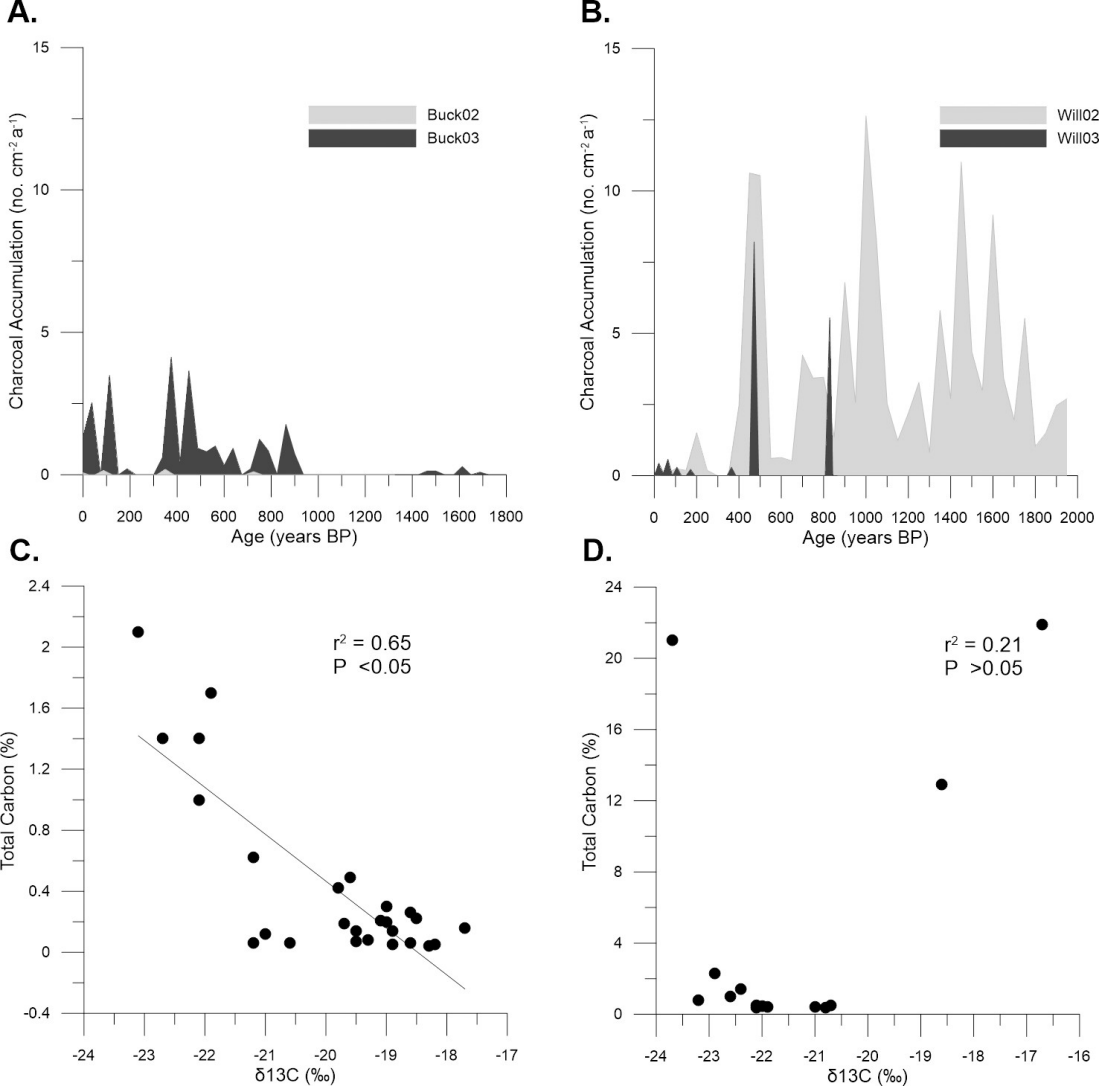
Macro-charcoal accumulation rates versus age and relationship between δ^13^C and total carbon. Demonstrates: (A) Buck02 and Buck03, and (B) Will02 and Will03. Where ages were calculated using a linear sedimentation rate for each core (see Table 2), (C) and (D) the relationship between δ^13^C and total carbon for (C) Will03 and (D) Buck03.

Therefore, based on the three acceptable OSL ages from the Buckiinguy cores, linear age-depth models provided mean vertical sedimentation rate estimates of 0.04 cm a^-1^ for Buck02 and 0.04 cm a^-1^ for Buck03 (Table 2, S2 Fig). These equate to bulk mass accumulation rates of 0.07 g cm^-2^ a^-1^ for Buck02, and 0.06 g cm^-2^ a^-1^ for Buck03. CHAR in Buck02 ranged from 0.07 to 0.19 no. cm^-2^ a^-1^, with a mean of 0.01 no. cm^-2^ a^-1^ over the last ~0.9 ka, with peaks in CHAR at 0.087, 0.35, 0.72 ka (Fig 9A). CHAR in Buck03 ranged from 0.03 to 3.75 no. cm^-2^ a^-1^, with a mean of 0.38 cm^-2^ a^-1^ over the last ~1.7 ka, and peaks in CHAR occurred at 0.03, 0.11, 0.37, 0.45 ka (Fig 9A). All age estimates carry relatively large, but unquantified, uncertainties. When assessing the CHAR records in relation to background flux, to determine autochthonous and allochthonous contributions, it is clear that flux is low and does not affect CHAR peaks.

**Table 2:** Summary of sediment accumulation rate, mean DBD, and bulk mass accumulation for Buck02 and Buck03, and Will02 and Will03.

Core	Location	Mean sedimentation rate (cm a^-1^)	Mean DBD (g cm^-3^)	Bulk mass accumulation rate (g cm^-2^ a^-1^)	Mean charcoal concentration (no. cm^-3^)	Mean CHAR (no. cm^-2^ a^-1^)
**Buck02**	Peripheral swamp	0.04	1.55	0.07	12.57	0.01
**Buck03**	Central swamp	0.04	1.51	0.06	26.24	0.38
**Will02**	Peripheral swamp	0.03	1.63	0.06	128.88	2.91
**Will03**	Peripheral swamp	0.07	1.61	0.11	7.81	0.23

The age of buried sediment in Will02 at a depth of 20–30 cm was 0.24 ± 0.07 ka, while a second sample from 40–50 cm yielded no useable data due to the lack of sufficient grains to make a single grain disk (Table 1, S1 Fig). The OSL ages determined for Will03 were 1.15 ± 0.11 ka at 40–50 cm and 32.54 ± 3.05 ka at 107–117 cm. The two acceptable OSL ages from the Willancorah cores, linear age-depth models provided mean vertical sedimentation rate estimates of 0.03 cm a^-1^ for Will02 and 0.07 cm a^-1^ for Will03 (Table 2, S2 Fig). These equate to bulk mass accumulation rates of 0.06 g cm^-2^ a^-1^ for Will02, and 0.11 g cm^-2^ a^-1^ for Will03. CHAR in Will02 ranged from 0.06 to 12.09 no. cm^-2^ a^-1^, with a mean of 2.91 no. cm^-2^ a^-1^ over the last ~1.9 ka and peaks at 0.45, 1.0, 1.45 and 1.6 ka (Fig 9B). CHAR in Will03 ranged from 0.21 to 8.33 no. cm^-2^ a^-1^, with a mean of 0.23 no. cm^-2^ a^-1^ over the last ~1.2 ka and peaks at 0.71 and 0.83 ka (Fig 9B).

The chronologies from Buckiinguy and Willancorah Swamps warrant some caution, due to the fact that only a limited number of quartz grains could be extracted from the samples because these are a mud dominated environments. Buck03 age 0.98 ± 0.15 ka at the sample depth of 75–85 cm resulting age estimate is younger than the age above 1.77 ± 0.21 ka with a 14.0% acceptance rate. Will03 age 32.54 ± 3.05 ka at the sample depth 107–117 cm only had 9.0% acceptance rate and was clearly an order of magnitude different from the other age (1.15 ± 0.11 ka) at the top of the core. For these reasons these ages from Buck03 and Will03 the ages were not included in the age-depth model (S2 Fig) as they are not a reliable representation of the grain population.

### Carbon stable isotopes

Buck03 δ^13^C signatures ranged from -23.7 to -16.7 ‰ in the upper 12 cm of the profile, and then changed slightly to -22.9 to -20.9 ‰ as total carbon decreased with depth below 15 cm (Fig 6F). In keeping with the organic rich sediment in the upper 15 cm of Buck03, total carbon was highest (21.9%) in this part of the profile and declined to <1% for the rest of the core.

Will03 δ^13^C signatures were more variable throughout the core profile, being close to -22.5 ‰ in the upper 10 cm of the profile and then enriching from -19.7 to 17.7 ‰ from 20 to 52.5 cm. A depletion to -22 ‰ was observed from 52.5 to 62 cm where there was also a slight increase in total carbon (up to 1.4%), followed by variations around -20 ‰ in the lower part of the core (Fig 8F).

Overall, there was a significant negative linear relationship (r^2^ = 0.65; p<0.05) between δ^13^C and total carbon in Will03 (Fig 9C), but no significant relationship (r^2^ = 0.21; p>0.05) in Buck03 due to three pronounced outliers (Fig 9D).

### Geochemistry

Magnetic susceptibility was variable but generally increased with depth in Buck02 and Buck03. This did not correspond with any change in sedimentology, organic matter, or macro-charcoal peaks (Figs 5F and 6G). Similarly, magnetic susceptibility was highly variable in both Will02 and Will03 cores, but the peaks and troughs did not align with any change in sedimentology, organic matter, or macro-charcoal peaks (Figs 7F and 8G).

Some Itrax elements were substituted by proxy for grain size (e.g., K, Ti, Si, Zr, and Fe) [84], and revealed no distinctive patterns that could be correlated with any sedimentary change in depth (S3 Fig). However, there was a noticeable decline in K, Ti, Si, Zr, and Fe proxies in Buck03 (S3 Fig) at depths of ~20 cm and ~34 cm, respectively. Itrax ratio results (S4 Fig) show variable trends throughout all profiles. Fe/Ti were used as indicators of smaller grain-size fluctuations from allochthonous material. Fe/Mn were used to assess reducing conditions (redox). Ca/Fe and Ca/Ti ratios suggest pedogenic processes, drier conditions, or biogenic concentration of Ca [85, 86]. Buck03 had a notable correlation between Ca/Ti and charcoal peaks at 15 cm, 20 cm, and 35 cm (S4 Fig). In Buck02, Fe/Ti and most other ratios were variable throughout the core (S4 Fig). Will02 and Will03 were also highly variable in Ca/Ti and Ca/Fe ratios (S4 Fig).

Principle Component Analysis (PCA) revealed some clustering of detrital and pedogenic elements in Buck02 and Buck03, for example, Ti, K, Si, Fe, Rb, and Ca (S5 and S6 Figs). PCA for Will02 also reveals a cluster of detrital elements of Fe, Ti, K, Si, and Zn, whereas Will03 PCA has a similar spread of detrital elements (S7 and S8 Figs). However, it is difficult to link PCA with the geochemistry results to variations in macro-charcoal or macro-charcoal peaks. Stratigraphic profiles of Buck02 and Buck03 (S5 and S6 Figs) with coloured bands representing geochemical groups based on the PCA plot, reveal a combination of detrital and pedogenic elements. Buck02 has a natural break in the geochemical profile at a depth of ~15 cm suggesting there were two different geochemical groups, whereas Buck03 has a similar break at a depth of ~15 cm. Will02 (S7 Fig) showed a natural break at a depth of ~22 cm in the stratigraphic profile, while Will03 revealed breaks at depths of ~30 cm and ~55 cm based on the geochemistry (S8 Fig).

## Discussion

### Allochthonous and autochthonous macro-charcoal sources and accumulation

The baseline estimate of allochthonous macro-charcoal flux (1.05 ± 0.32 no. cm^-2^ a^-1^) derived from contemporary fluvial sediment deposited in Buckiinguy Swamp indicates that charcoal entering the system from upstream is one confounding factor for fire history reconstruction in the Macquarie Marshes and similar open-system wetlands. Although it is often assumed that all macro-charcoal (>125 um) found in stable depositional environments is autochthonous, our results show that a small but important amount of macro-charcoal is allochthonous. This has been recognised previously, for example, where macro-charcoal contributions to boreal lakes occur from both local and regional sources [22]. Indeed, the source areas of charcoal (e.g., catchment and/or river corridor) and the distance travelled tend to be difficult to disentangle, especially in large catchments where wetlands and floodplain pockets can act as filtration traps for charcoal and sediment [47, 87]. By identifying the allochthonous macro-charcoal signal and taking this into account when assessing sediment cores, autochthonous macro-charcoal records in sediment were derived for the first time in this type of open-system wetland.

Within Buckiinguy Swamp, flow attenuation by dense vegetation and differential patterns of overbank and floodout sedimentation led to variations in the volume of fluvially-derived sediment and macro-charcoal deposited on the floodplain surface and in the wetlands (Figs 3 and 4). While there was no correlation between deposited sediment volume and macro-charcoal, site B3 in the floodout zone (closest to the channel terminus) captured the greatest volume of sediment (Fig 3A) when there was a rapid decline in channelised flow and an increase in sheet flow, with a dominance of very low-energy conditions. This is common in low-relief floodplain wetlands where channel breakdown occurs and where in-channel and wetland vegetation blocks the flow and distributes water and sediment onto the floodplain [35, 47], thereby affecting sediment and charcoal distribution. Site B4 exhibited the greatest mean macro-charcoal concentration and flux, the latter within the standard error of the other sites, possibly due to local topographic variation, reworking, or concentrated deposition through other processes (e.g., back-flooding caused by flow-attenuation near the outlet of the swamp). Nevertheless, floodplain wetlands act as sinks for sediment, nutrients, and contaminants from upstream [62, 88]. In this case, the allochthonous contribution and spatial variation of macro-charcoal and sediment entering the system from upstream are influenced by the vegetation, irregular surface topology, and hydrodynamic characteristics of flow in the wetlands. Whether this redistribution and deposition results in permanent storage of macro-charcoal is unknown. In addition, alternative sources of macro-charcoal such as wind-blown particles carried aloft from ground or crown fires in the wetlands (or elsewhere) are another factors that could contribute to the charcoal pool, but this is unable to be quantified in this study. It may also be that other feeder channels entering the wetlands carry different macro-charcoal loads, due to their intermittent and/or ephemeral flow regimes.

The autochthonous macro-charcoal records found in cores from Buckiinguy and Willancorah Swamps were highly variable with depth (despite the sedimentology being similar), yet there were inconsistencies between cores within each wetland, and between the two wetlands, beyond the limits imposed by the age models. Will02 had by far the greatest macro-charcoal accumulation and numerous large peaks throughout the profile (Fig 7), despite the recent history of fire in Willancorah which showed just 6 ignition points from 2002–2016 (Fig 2B). Macro-charcoal found at depth in Willancorah is therefore present due to fire activity preceding the period of availability of Sentinel Hotspot information. Will03 had just two distinct peaks at depths of 34.5 cm and 59 cm, with very little macro-charcoal elsewhere in the profile (Fig 8). This highlights the variability of fire activity and/or macro-charcoal deposition in the wetland over space and time, and the long-term macro-charcoal record yields very different results to the short-term satellite record. Nevertheless, Willancorah has lower concentrations of macro-charcoal at the surface, in keeping with the low number of recent ignitions as evidenced by Sentinel Hotspot. Conversely, Buck03 (Fig 6) had relatively high macro-charcoal concentration and flux at the surface. Correspondingly the wetland had 33 ignition points from 2002–2016, with several high-confidence hotspots occurring near the sediment coring sites, with no wildfire or prescribed burns in or around the wetland (Fig 2C). Despite this positive correlation between fire ignition points and charcoal at the surface of Buck03, there was very little macro-charcoal accumulation at Buck02. Overall Buckiinguy had less macro-charcoal accumulation than Willancorah in the long-term record.

The long-term macro-charcoal record for Buckiinguy Swamp is constrained by OSL dating to the last ~1.7 ka in Buck03 (Fig 6), where consistently higher charcoal concentrations exist in the upper 40 cm of the profile. Four CHAR peaks (2.5–4 no. cm^-2^ a^-1^) occur at 0.05, 0.11, 0.37, and 0.45 ka (Fig 9A). Prior to ~1 ka, there is little charcoal preservation. Buck02 has very little macro-charcoal throughout (Fig 5), but the record is constrained to the last ~1 ka where there are three very minor CHAR peaks (<1 cm^-2^ a^-1^) at 0.02, 0.35, and 0.72 ka (Fig 9A). Common peaks between Buck02 and Buck03 were difficult to discern, bearing in mind the limits of the age models due to the limited OSL samples, and the fact that a zero age was applied to the surface (i.e., 0 cm) to calculate mean linear sedimentation rates for the cores. Our sediment accumulation rate estimates (from 0.03–0.07 cm a^-1^) do concur with previous results from Ralph *et al*. [46] who derived values of 0.032–0.037 cm a^-1^ in the same wetlands [46, 47]. The variation between these two cores indicates highly variable fire activity within Buckiinguy Swamp, with either more frequent, or more intense, fires and/or greater macro-charcoal deposition in the densely vegetated centre of the reed bed (Buck03) as opposed to the margin of the reed bed (Buck02). Another possibility is that charcoal deposition is uneven over short distances within the reedbeds, either because of uneven distribution of allochthonous macro-charcoal or reworking (by flows within the marshes) shortly after fire events.

The long-term macro-charcoal record in Willancorah Swamp was constrained in Will02 mid-way in the profile to ~0.24 ka, and at Will03 to the last ~1.15 ka (Figs 7 and 8). These cores had highly variable charcoal counts with little similarity to the Buckiinguy cores, or each other although they were only taken ~17 m apart at the margin of the swamp. Will02 had large charcoal peaks scattered throughout the core (~5 to 60 cm) with CHAR up to 12 cm^-2^ a^1^ indicating a greater frequency or intensity of fire over the past ~0.24 ka (Fig 9B). Will03 had two large CHAR peaks (>5.55 cm^-2^ a^-1^) at 0.47 and 0.83 ka indicating isolated or irregular fire activity within the last ~2 ka (Fig 9B).

A much older OSL age obtained at the base of Will03 is unreliable (S1 and S2 Figs) due to the very small acceptance rate of grains (9.0%) and therefore was not used to calculate CHAR. This anomalous age could be due to partial bleaching during sediment transport and deposition, which prevents the complete resetting of the luminescence signal and is common in fluvial systems [89, 90]. Since the partial bleaching of grains is common in fluvial systems, the minimum age model (MAM) was applied to all single-grain OSL data to isolate the grains with the smallest residual dose to minimise any age overestimation [78, 79]. The marshes formed over the last ~5.5 ka [91], so the ~33 ka age at the base of Will03 is beyond the maximum age for the marshes and, if real, could represent the age for the underlying palaeo-floodplain which is in keeping with OSL chronologies for the surrounding palaeochannels [92, 93]. Despite this, CHAR was calculated for the upper part of the profile using the other OSL age, peaks occurred at 0.47 and 0.83 ka. There was a common CHAR peak that noticeably stood out at ~0.47 ka between Will02 and Will03 (Fig 9B).

### Interpretation of δ^13^C signatures and geochemistry

Carbon stable isotope analysis can help disentangle vegetation shifts in the environment over time as well as the interpretation of past environmental conditions [94, 95]. The discrimination of ^13^CO_2_ by plants through diffusional and biochemical reactions from carbon fixing enzymes can help assist in separating different types of photosynthetic groups [96]. C3 plants tend to have depleted values ranging from -40 to -20 ‰ typically constituting woody species, whereas C4 plants consist of mostly grasses with enriched values of -17 to -9 ‰ [94, 97]. The C4 plant water couch (*Paspalum distichum*) has a typical δ^13^C value of -18.7 ‰, while the C3 plant common reed (*Phragmites australis*) has typical values of -28.2 to -27.6 ‰ [59, 98 p. 90] (S1 Table). The δ^13^C profile from Buck03 suggests a C4 signature in the upper 10 cm, while below 10.5 cm, δ^13^C values indicate a C3 signature (Fig 6F). In contrast, Will03 revealed a possible mixture of C3 and C4 vegetation throughout the core as the values remain close to -20 ‰, although there appears to be a shift towards C3 species at 59–62 cm (Fig 8F). It is worth noting that the factors which affect δ^13^C values in wetlands and which can complicate interpretations of carbon source and C3 or C4 vegetation [96, 99, 100], include bacteria, algae, sugars, starch in organic matter, and isotope fractionation.

Total organic carbon decreased in the cores with increasing depth and had a negative relationship with δ^13^C in Buck03, indicating that the storage of carbon in the wetlands is minimal and is restricted to the upper organic-rich at a depth of ~10 cm of the cores. This was observed in a previous study of carbon in the Macquarie Marshes [59]. Overall, it is not possible to interpret changes in δ^13^C and total organic carbon results (Figs 6F and 8F) or to extend these to relate to vegetation succession and/or fires as this is beyond the scope of the study.

Magnetic susceptibility results from Buckiinguy and Willancorah were inconsistent and did not show any step-changes or correlations with sedimentology or macro-charcoal peaks (Figs 5–8). Magnetic susceptibility was used as a proxy to help distinguish between sources of magnetically transported allogenic clastic minerals in lakes [101–104]. Fire can remove surface coverage of vegetation and strip the ground clear leaving it susceptible to erosional processes [102]. Magnetic susceptibility coupled with charcoal records could possibly help in an attempt to discriminate the allochthonous input of minerogenic material from fire events [19, 105]. Millspaugh and Whitlock [105] and Long *et al*. [19] attempted to use magnetic susceptibility and charcoal results to assess the role of fires as triggers for erosional events. However, in their work, only some charcoal peaks were correlated with magnetic susceptibility peaks suggesting that fire is not the only disturbance agent to instigate erosional processes. Magnetic susceptibility in a system like the Macquarie Marshes could vary due to sediment particle size, pedogenic nodules of iron or manganese, or more complex processes related to the input of sediment and river discharge that govern the system.

As previously noted some elements (e.g., K, Ti, Si, Zr, and Fe) were used to infer sediment grain size from Itrax core scanning in other studies [84, 106]. Sedimentary profiles with Fe and K are associated with clay-rich layers, Si and Zr with sandy coarse silts, and Ti with silts [84]. However, selected proxies for grain size analysis did not reveal any significant changes in grain size correlating with sedimentary changes in our cores (S3 Fig). The exception is Buck03 core where there were two distinct declines in selected elements (K, Ti, Si, Zr, and Fe) respectfully at ~20 cm and ~35 cm, this sharp decline is most likely due to organic material (i.e., plant root at ~20 cm) and minor split in the core (S3 Fig).

The elemental ratios derived from Itrax for sediment profiles in Buckiinguy and Willancorah Swamps were highly variable but did not show step-changes and could not clearly differentiate detrital inputs from *in situ* products of soil formation (i.e., pedogenic processes) (S4 and S5–S8 Figs). In other systems, ratios of geochemically stable elements have been used to track chemical or physical weathering in catchments and as indicators of detrital inputs [107]. Titanium (Ti) is used more readily than other elements (e.g., iron, Fe) as a reliable indicator of detrital input as Fe and other elements can be affected by reduction and oxidation processes [108]. Variations in grain size combined with geochemical data (e.g., Fe/Ti) of allochthonous material in lake sediment has been used to infer different types of energy conditions at the time of deposition and the nature of the derived material [108, 109]. Buck03 is the only core that has two distinct CHAR peaks that correlate with Itrax data for Fe/Ti coinciding with Fe-rich clastic material suggesting detrital input or a fire exposing bare soil (S4 Fig). Haberzettl *et al*. [110] used Ca/Ti ratios to infer hydrological variability where high values indicate drier conditions and low values indicate wetter conditions. Buck03 has increases in Ca/Ti at ~20 cm and ~35 cm that seem to correspond with an increase in macro-charcoal concentration, which may be related to drier conditions and coincides with higher sand content suggesting the possibility of increased fluvial deposition of charcoal and sediment together. Will02 at ~52 cm also has a spike in Ca/Ti (S4 Fig); however, this is not seen in all cores, since Will03 has no Ca/Ti peak at 35 cm corresponding with a charcoal peak, nor does Buck02 have matching peaks (except for ~15 cm). Will03 at ~55 cm has a break prior to the start of the second core and is most likely due to contamination. PCA and stratigraphic (S5–S8 Figs) analysis indicate that there are natural breaks in the cores geochemically, but they do not reveal any significant trends. Overall, both PCA and stratigraphic analysis of cores from Buckiinguy and Willancorah Swamps showed highly variable results with no significant trends or correlations between the records.

### Problems and prospects for reconstruction of fire regimes and environmental conditions in floodplain wetlands

Investigations of fire activity in large, open-system wetlands in dryland regions have been few and far between [36, 111, 112]. For example, fire has been studied in the large (~12,000 km^2^), dynamic wetlands of the Okavango Delta, Botswana using satellite imagery, but macro-charcoal was not utilised as an indicator of fire activity [111–113]. The Sudd in southern Sudan (~40,000 km^2^) is another significant inland wetland system susceptible to fire, however again there are very sparse fire and associated environmental records [114, 115]. Most palaeo-fire studies examine and interpret macro-charcoal from one or two sediment cores at high resolution [7, 8, 25, 116, 117]. Commonly, this is done in small, closed-system lakes or wetlands due to their propensity to accumulate and preserve charcoal and sediment over time. There has been a limited of number studies that attempt to cross-correlate charcoal records between cores within wetlands, or to confirm the reproducibility of down-core patterns of charcoal concentration [87]. In this study, we attempted to correlate charcoal records from two key wetlands in a large, dynamic, open-system; however the results were difficult to interpret and indicate that charcoal accumulation, fluvial supply of charcoal, and wetland fire regimes are highly variable in space and time.

Any attempt to reconstruct fire regimes in large wetlands and/or catchments may be difficult due to the issues associated with using macro-charcoal of unknown or variable origin as an indicator of local fire history. The major problems facing fire history reconstruction using macro-charcoal records in large, dynamic, open-system wetlands include: (1) large spatial and temporal variations in fire activity, ash and charcoal products within the wetlands, (2) large variations in inputs of allochthonous charcoal from various upstream sources, (3) high likelihood of geomorphic dynamism affecting flow dispersal and sediment and charcoal accumulation, and (4) propensity for post-depositional reworking, modification and/or destruction of macro-charcoal by floods and taphonomic processes. Importantly, human activity affects these for factors in different ways, and can be viewed as an additional layer of impact that would need to be quantified in any future research.

The results of this study indicate that there are significant implications for any future reconstruction of macro-charcoal records in large, dynamic, open-system wetlands. Firstly, spatial and temporal variations in fire activity and ash and charcoal products within the wetlands are important because they generate an uneven distribution of charcoal in these wetland systems. Wetlands that experience fires do not burn uniformly and this is due to the local environmental conditions (e.g., ignition mechanism, flammability, vegetation type, and fuel production) and climatic and weather controls (e.g., rainfall, water balance, wind, temperature) that influence the ignition and spread of fires [118–120]. For example, research has shown fires were absent from some permanent wetlands in semi-arid north-eastern South Africa [120]. Conversely, fire is a common occurrence in some seasonal wetlands, as they are more susceptible to fire activity due to the drying of vegetation during the winter months and the hydrological regime [120]. Research examining the spread of fires (following a prescribed burn) across savanna-wetland ecotones also showed different burning patterns between herbaceous and ligneous vegetation types [118]. Herbaceous plants (i.e., non-woody) tend to undergo full combustion leaving no residual macro-charcoal, whereas their ligneous counterparts (i.e., woody) produce a majority of macro-charcoal in the sedimentary record [121–123]. This complicates the spatial and temporal patterns of fire activity and contributes to the unevenness of the distribution of charcoal across a wetland after a fire event. Understanding the detailed patterns of vegetation, fuel availability, soil carbon and water balances in wetlands could assist in the development of fire management practice in the future.

Secondly, varying inputs of allochthonous charcoal from various upstream sources are problematic because interpreting local fire history relies on the assumption of primary charcoal being rapidly deposited and buried during and immediately after a fire event; any charcoal incorporated later is regarded as being from a secondary source or from non-fire years [15]. While we did not determine the source/s of allochthonous macro-charcoal in the Macquarie Marshes, there is potential for it to have come from the catchment (e.g., relatively young particles in topsoil), or from reworking of material along the river corridor (e.g., relatively older particles from subsoils and river banks). Nevertheless, our findings illustrate that macro-charcoal inputs from other sources in the upstream catchment are a significant component deposited in the open-system wetlands of the Macquarie Marshes during non-fire years. Altogether, catchment conditions, geomorphic setting and processes, climate and hydrology, ignition sources, and readily burnable biomass can all affect the transport, deposition, and taphonomy of charcoal in a sediment profile [20, 31, 124].

Theoretical models of charcoal dispersal predict that charcoal particle size should decrease with distance from the burn area [21, 125]. Research shows that allochthonous macro-charcoal can remain in suspension for a longer duration in comparison to smaller charcoal particles [126]. Nevertheless, it is likely that in a large catchment with a subhumid to semiarid climate such as the Macquarie, macro-charcoal distribution occurs sporadically during seasonal flows, infrequent large floods, or rainfall events. However there have been a limited number of empirical studies validating this, through either natural or prescribed burns using traps within proximity of the burn (e.g., <200 m) to collect charcoal and track dispersal patterns [16, 123, 127, 128]. Tracking macro-charcoal dispersal patterns in catchments and determining the major sources and pathways of transport and storage in large river and wetland systems would be an immense undertaking that could potentially yield a greatly enhanced understanding of charcoal dynamics in fluvial systems. This could assist in the future analysis of macro-charcoal in open-system wetlands.

Thirdly, geomorphic dynamism affects flow dispersal and sediment and charcoal accumulation patterns in floodplain wetlands. The complexity of landforms and geomorphic processes that control the distribution of water and sediment in these wetlands plays an important role for the interpretation and understanding of sedimentary macro-charcoal and fire records. Anabranching and distributary channels that feed water into wetlands such as the Macquarie Marshes [35, 47], the Okavango Delta [129], and the Sudd [114] distribute macro-charcoal from upstream, and may rework previously buried charcoal through erosion processes within channels and on the floodplain, leading to new and/or multiple foci of charcoal accumulation. Reticulate surface drainage patterns, low levees, flood retention zones, floodouts, and gilgai (i.e., depressions and low mounds) also influence dispersal of water, sediment, and charcoal in these systems [47]. These geomorphic units are a function of channel and floodplain erosion, sedimentation and post-depositional processes such as clay shrink-swell behaviour that also affect charcoal supply, distribution, deposition, and reworking. While overbank flooding is a critical driver of wetland vegetation and habitat for various aquatic and terrestrial species, changes in rainfall, evapotranspiration, and flood regime variability are likely to influence channel patterns and thus macro-charcoal accumulation patterns [46, 130]. Channel erosion leading to a loss of channel-floodplain connectivity may exacerbate the risk of major fires across the system due to reduced surface water distribution to the contemporary wetlands. Characterising the landforms and processes responsible for geomorphic dynamism and flow distribution in wetlands is critical for assessment of the role of these processes in macro-charcoal accumulation and for fire history reconstructions.

Finally, post-depositional reworking, modification and/or destruction of macro-charcoal by floods and/or taphonomic processes are major problems in wetlands in drylands that flood irregularly and dry out regularly. Large or energetic floods have the potential to rework sediment and charcoal in wetlands. Taphonomic processes of physical, biological, and chemical nature could also affect charcoal preservation, including disturbance and/or erosion of topsoils, bioturbation (mixing) by ants and other burrowing organisms in the upper sections of profiles [131], and decomposition and/or degradation of charcoal and other organic matter under varying anoxic and oxidising conditions [132, 133]. In particular, oxidation of charcoal and the overall loss of susceptible charcoal particles to biochemical degradation could potentially lead to an underestimation of macro-charcoal used as a proxy for fire regime reconstruction [132, 133]. Soil and sediment erosion, trampling impact of livestock and other animals, a suite of activities related to agriculture, and other human activities can also alter sediment records. Livestock are known to spend a significant amount of time around wetlands where they trample and churn the topsoil [64, 134, 135]. For this reason, knowledge of site, system history, and careful site selection is essential to avoid sampling disturbed sediments. Furthermore, the inherent behaviour of vertisol soils [47, 136] can influence the preservation of charcoal, where the physical shrink-swell properties of clay soils in response to sporadic wetting and drying cycles could impact the distribution of charcoal in the profile (i.e., self-mulching clay soils). The physical, biological, and chemical processes that govern taphonomy and the propensity for deposition and reworking of charcoal in open-system wetlands should be understood and interpreted with care, while considering the other environmental and geomorphic conditions previously mentioned.

Looking to the future, we must acknowledge that large, open-system floodplain wetlands have complex and highly variable macro-charcoal records that defy simple interpretations and straightforward fire history reconstructions. This study quantified the mean allochthonous macro-charcoal supply for one wetland in the Macquarie Marshes, and then extrapolated this to another wetland in the same system. While this method can help to discriminate the allochthonous macro-charcoal signal which can then be subtracted from total charcoal counts to obtain corrected autochthonous macro-charcoal profiles in sediment cores, a better approach would be to quantify the fluvial input to each individual wetland through a long-term monitoring program. Furthermore, in the sediment profiles, higher sensitivity and resolution sedimentological, geochemical and isotope analyses may be able to detect subtle changes in sediment and carbon character related to changing inundation regimes, which could be linked to changing macro-charcoal concentrations and/or taphonomic processes. Doing so would greatly improve the confidence around interpretations of macro-charcoal accumulation rates, carbon sources and fire history.

Determining whether fire activity contributes significantly to macro-charcoal accumulation in floodplain wetlands, and whether macro-charcoal of a local origin is useful for fire history reconstruction, is a complex task due to the nature of sediment and charcoal sources, transport and taphonomic processes in dynamic, open-system wetlands. For contemporary fire regime assessments, satellite remote sensing (e.g., Sentinel Hotspot information) combined with on-ground fire assessment and monitoring could be a powerful tool for wetland and water managers who need to understand fire activity, patterns, and regimes in floodplain wetlands in the future. Combining the use of satellite imagery with on-ground studies, and expanding on previous charcoal dispersal studies in smaller closed-system wetlands, could be an ideal prospect for future fire studies in open-system wetlands [21, 22, 123, 128, 137].

Assessing the longer-term history of fire in key wetlands based on reliable palaeo-environmental records could help guide future wetland management, especially when:

1. the sites are geomorphologically stable and are prone to fire in the recent and long-term past;
2. there is significant potential for preservation of macro-charcoal and other biological remains when their disturbance and degradation is minimal;
3. the links between fire, inundation regimes, and ecological processes are understood and valued for management purposes.

Ultimately, knowledge of historical fire regimes in wetlands over recent decades and during the historical period is critically important. Using sediment and macro-charcoal profiles to yield accurate and reproducible information can provide a longer-term context for understanding fire regimes.

## Conclusions

Understanding allochthonous and autochthonous contributions to macro-charcoal accumulation in open-system floodplain wetlands is critical for robust interpretation of fire history. The spatial and temporal patterns of fire activity are complex and deserve detailed study in terms of patterns of vegetation, fuel availability, soil carbon and water balances in wetlands. Tracking macro-charcoal dispersal patterns in catchments and determining the major sources and pathways of transport and storage in large wetland systems could potentially yield an improved understanding of charcoal dynamics in fluvial systems, including open-system wetlands. Characterising the landforms and processes responsible for geomorphic dynamism and flow distribution in wetlands is critical for assessment of macro-charcoal accumulation and for the reconstruction of fire history. Knowledge of site and system history, and careful site selection is essential to avoid sampling disturbed sediments that may obscure the interpretation of the macro-charcoal record. We have outlined the major problems and prospects for reconstruction of fire regimes and environmental conditions in large, dynamic, open-system wetlands. The application of macro-charcoal and other environmental proxy information in wetlands in drylands is inherently difficult due to variations in charcoal sources, sediment and charcoal deposition rates, and taphonomic processes. To reconstruct and interpret wetland fire regimes, recognition of complex fire-climate-hydrology-vegetation interactions is essential and high-resolution, multifaceted approaches are required to assess and understand spatial and temporal patterns of fire.

## Supporting information

S1 Data Manuscript data. (XLSX)

S2 Data Supplementary references for S1 Table. (DOCX)

S1 Fig Single-grain OSL radial plots. OSL radial plots: (A) Buck02 20–30 cm (n = 111), (B) Buck02 42–52 cm (n = 51), (C) Buck03 30–40 cm (n = 189), (D) Buck03 75–85 cm (n = 14), (E) Will02 20–30 cm (n = 78), (F) Will03 30–40 cm (n = 17), and (G) Will03 107–117 cm (n = 9).
(TIF)

S2 Fig Age-depth models. Illustrating the linear sedimentation rate for: (A) Buck02, (B) Buck03, (C) Will02, (D) and (E) Will03.
(TIF)

S3 Fig Selected elements to infer sediment grain size for Buck02, Buck03, Will02 and Will03. Selected elements are: K, Ti, Si, Zr, and Fe (blue shading represents selected charcoal peaks).
(TIF)

S4 Fig Geochemical ratios from ITRAX core scanning for Buck02, Buck03, Will02 and Will03. Selected ratios include: Fe/Ti, Fe/Mn, Ti/K, Zr/Rb, Ca/Fe, and Ca/Ti (Redline moving 10-point average, and blue shading represent selected charcoal peaks).
(TIF)

S5 Fig Stratigraphic profile and Principle component analysis (PCA) for Buck02. Demonstrates: (A) geochemical elements indicating similarities, and (B) PCA biplot showing concentrations of elements in groups based on geochemical grouping. Coloured numbers refer to the sample depth.
(TIF)

S6 Fig Stratigraphic profile and Principle component analysis (PCA) for Buck03. Demonstrates: (A) geochemical elements indicating similarities, and (B) PCA biplot showing concentrations of elements in groups based on geochemical grouping. Coloured numbers refer to the sample depth.
(TIF)

S7 Fig Stratigraphic profile and Principle component analysis (PCA) for Will02. Demonstrates: (A) geochemical elements indicating similarities and (B) PCA biplot showing concentrations of elements in groups based on geochemical grouping. Coloured numbers refer to the sample depth.
(TIF)

S8 Fig Stratigraphic profile and Principle component analysis (PCA) for Will03. Demonstrates: (A) geochemical elements indicating similarities and (B) PCA biplot showing concentrations of elements in groups based on geochemical grouping. Coloured numbers refer to the sample depth.
(TIF)

S1 Table Comparison of different values of δ^13^ C (‰) derived from C_3_ and C_4_ vegetation. Some selected stable isotope information of δ^13^ C (‰) from the literature for different types of vegetation located in Murray-Darling Basin in south-eastern Australia
(XLSX)
