# Supplementary material for: Macro-charcoal accumulation in floodplain wetlands: Problems and prospects for reconstruction of fire regimes and environmental conditions
Source: PLoS One. 2019 Oct 24;14(10):e0224011. doi: 10.1371/journal.pone.0224011 (PMC6812773; doi:10.1371/journal.pone.0224011)
Supplement: S2 Data — (DOCX) [file pone.0224011.s002.docx]

**References for S1 Data Table.**

138 - Yu L. Palaeoenvironmental change in the Macquarie Marshes, NSW, Australia. University of Wollongong, NSW, Australia. 2014.

139 - Kelleway J, Mazumder D, Wilson GG, Saintilan N, Knowles L, Iles J, et al. Trophic structure of benthic resources and consumers varies across a regulated floodplain wetland.
