# Supplementary figures and images for: Macro-charcoal accumulation in floodplain wetlands: Problems and prospects for reconstruction of fire regimes and environmental conditions
Source: PLoS One. 2019 Oct 24;14(10):e0224011. doi: 10.1371/journal.pone.0224011 (PMC6812773; doi:10.1371/journal.pone.0224011)

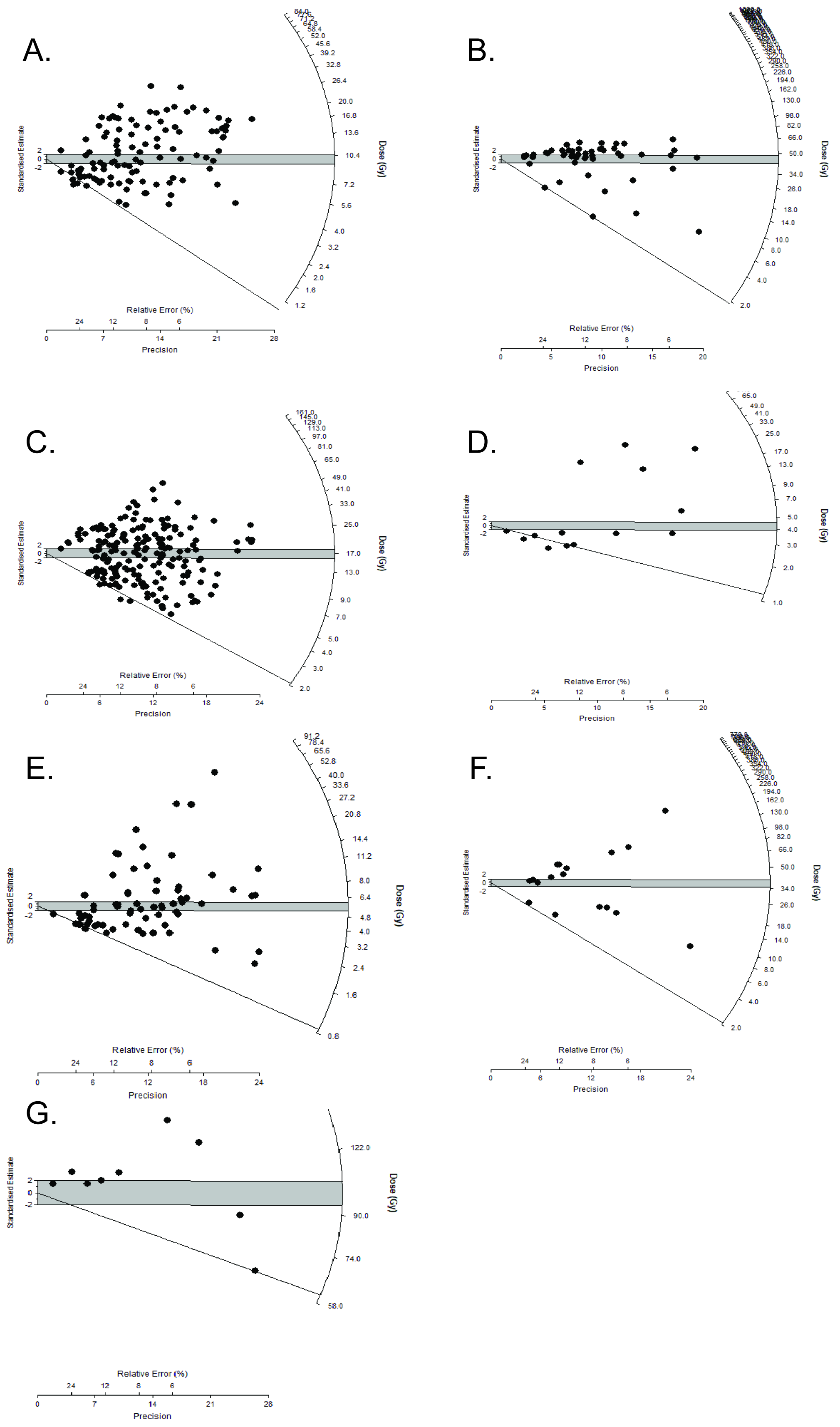

Supplement: S1 Fig — OSL radial plots: (A) Buck02 20–30 cm (n = 111), (B) Buck02 42–52 cm (n = 51), (C) Buck03 30–40 cm (n = 189), (D) Buck03 75–85 cm (n = 14), (E) Will02 20–30 cm (n = 78), (F) Will03 30–40 cm (n = 17), and (G) Will03 107–117 cm (n = 9). (TIF) [file pone.0224011.s003.tif]

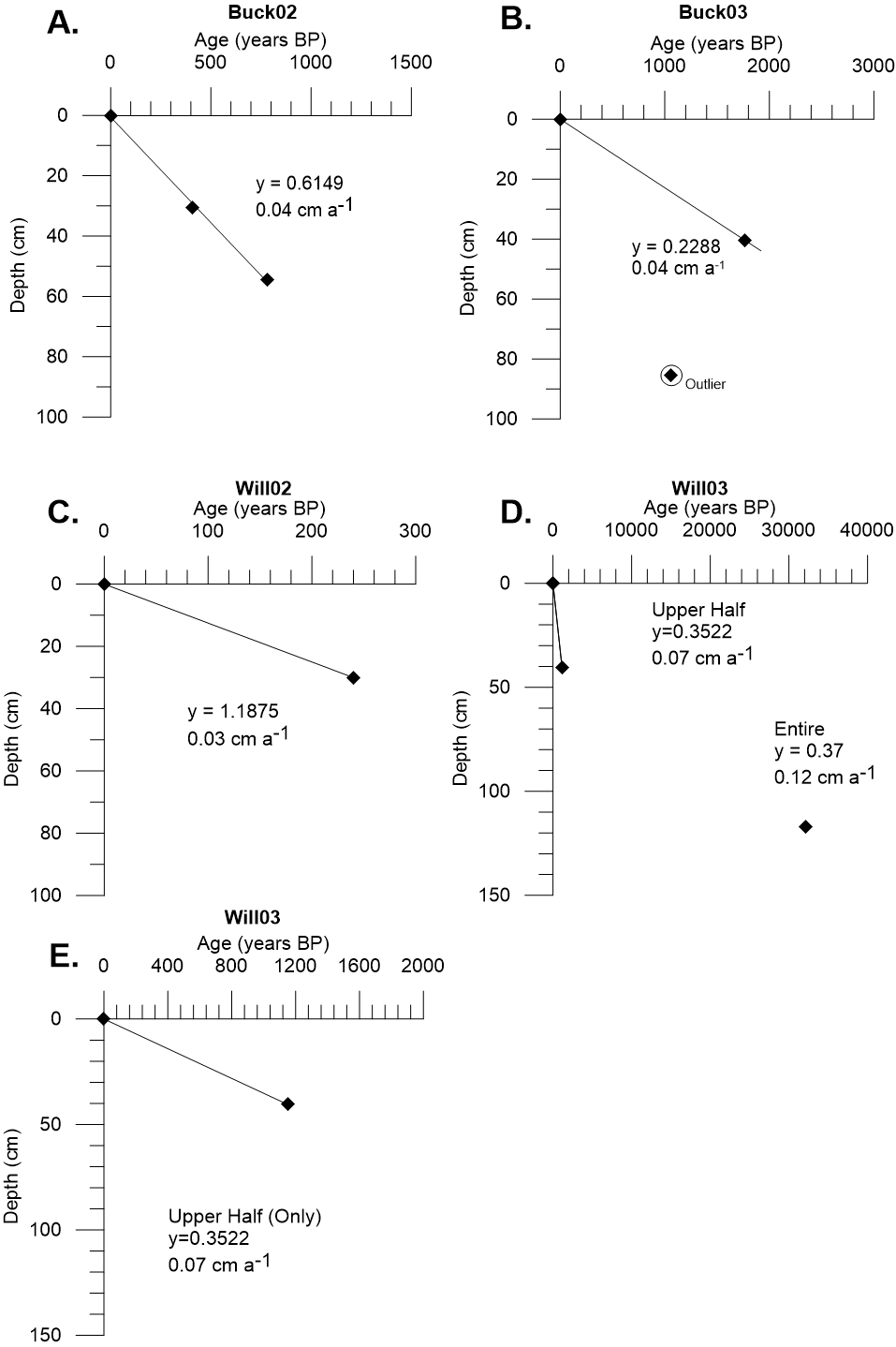

Supplement: S2 Fig — Illustrating the linear sedimentation rate for: (A) Buck02, (B) Buck03, (C) Will02, (D) and (E) Will03. (TIF) [file pone.0224011.s004.tif]

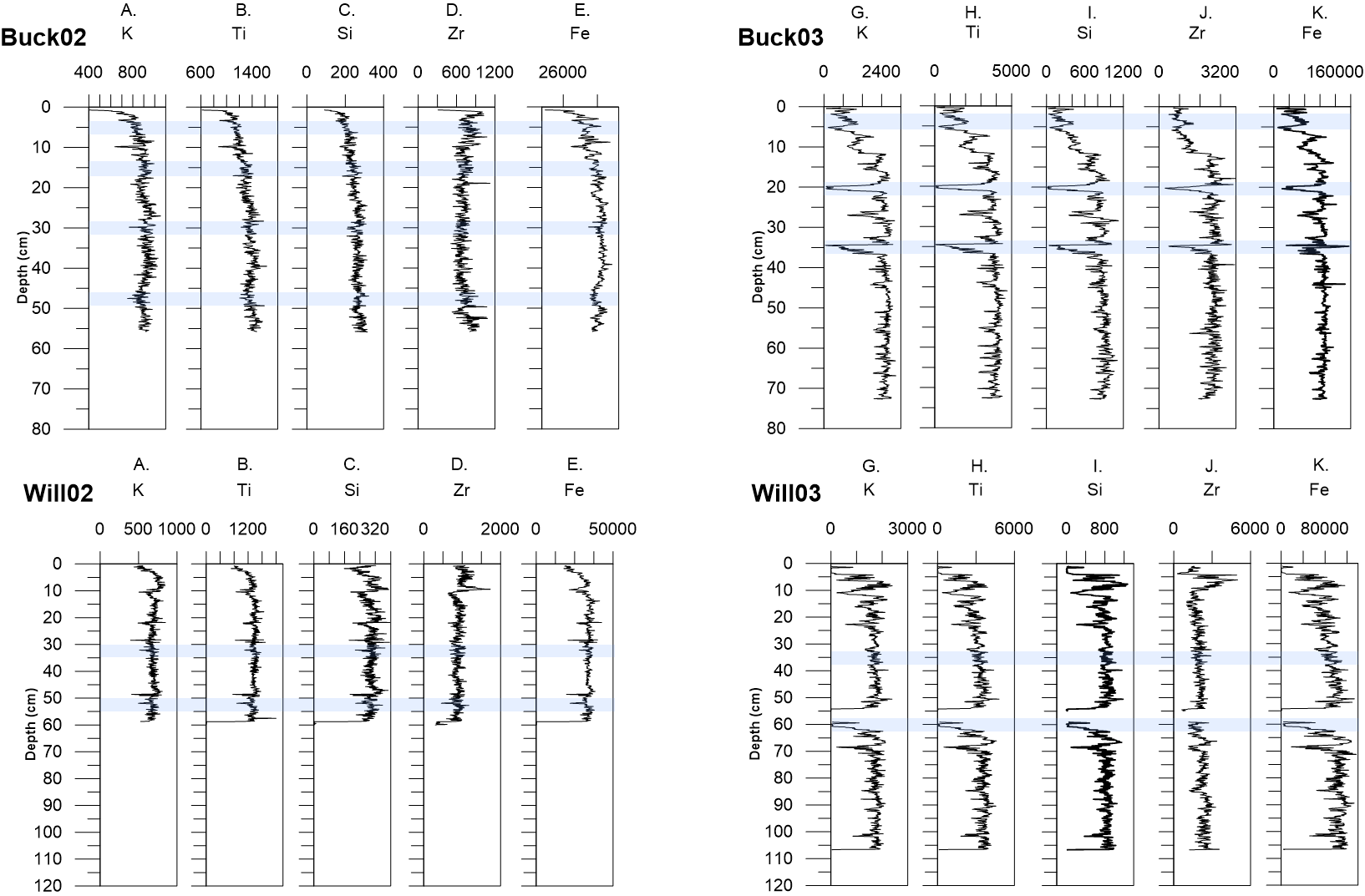

Supplement: S3 Fig — Selected elements are: K, Ti, Si, Zr, and Fe (blue shading represents selected charcoal peaks). (TIF) [file pone.0224011.s005.tif]

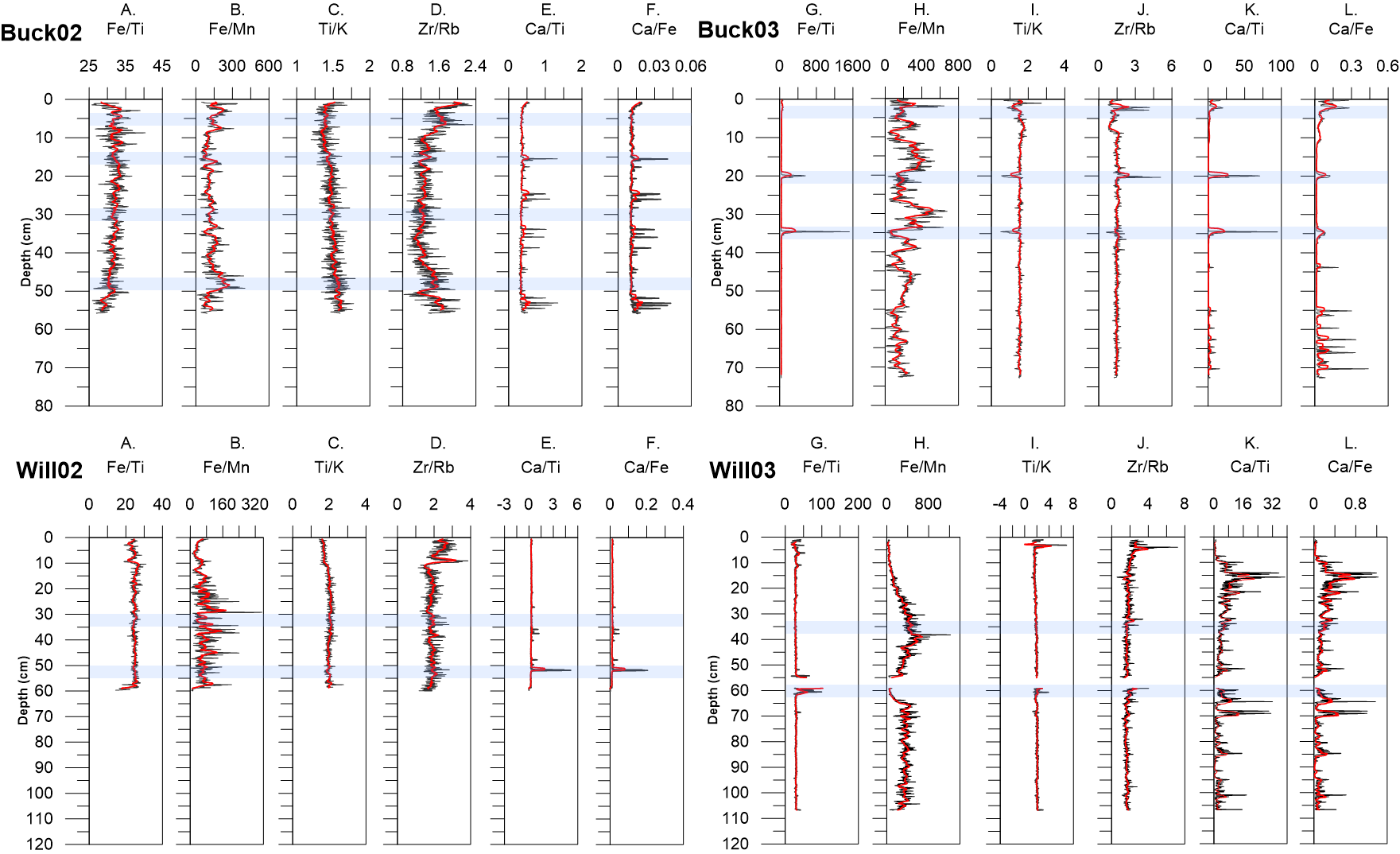

Supplement: S4 Fig — Selected ratios include: Fe/Ti, Fe/Mn, Ti/K, Zr/Rb, Ca/Fe, and Ca/Ti (Redline moving 10-point average, and blue shading represent selected charcoal peaks). (TIF) [file pone.0224011.s006.tif]

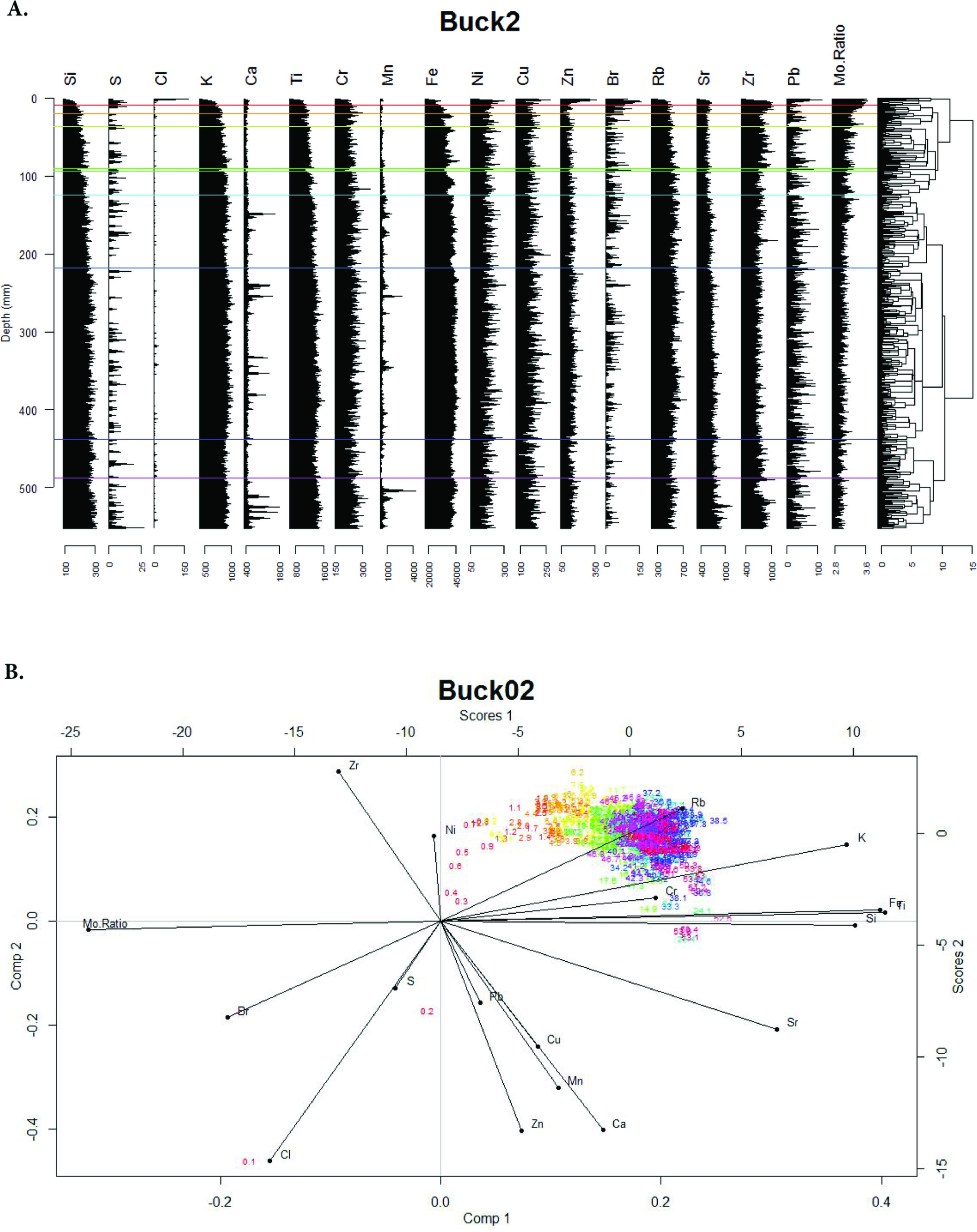

Supplement: S5 Fig — Demonstrates: (A) geochemical elements indicating similarities, and (B) PCA biplot showing concentrations of elements in groups based on geochemical grouping. Coloured numbers refer to the sample depth. (TIF) [file pone.0224011.s007.tif]

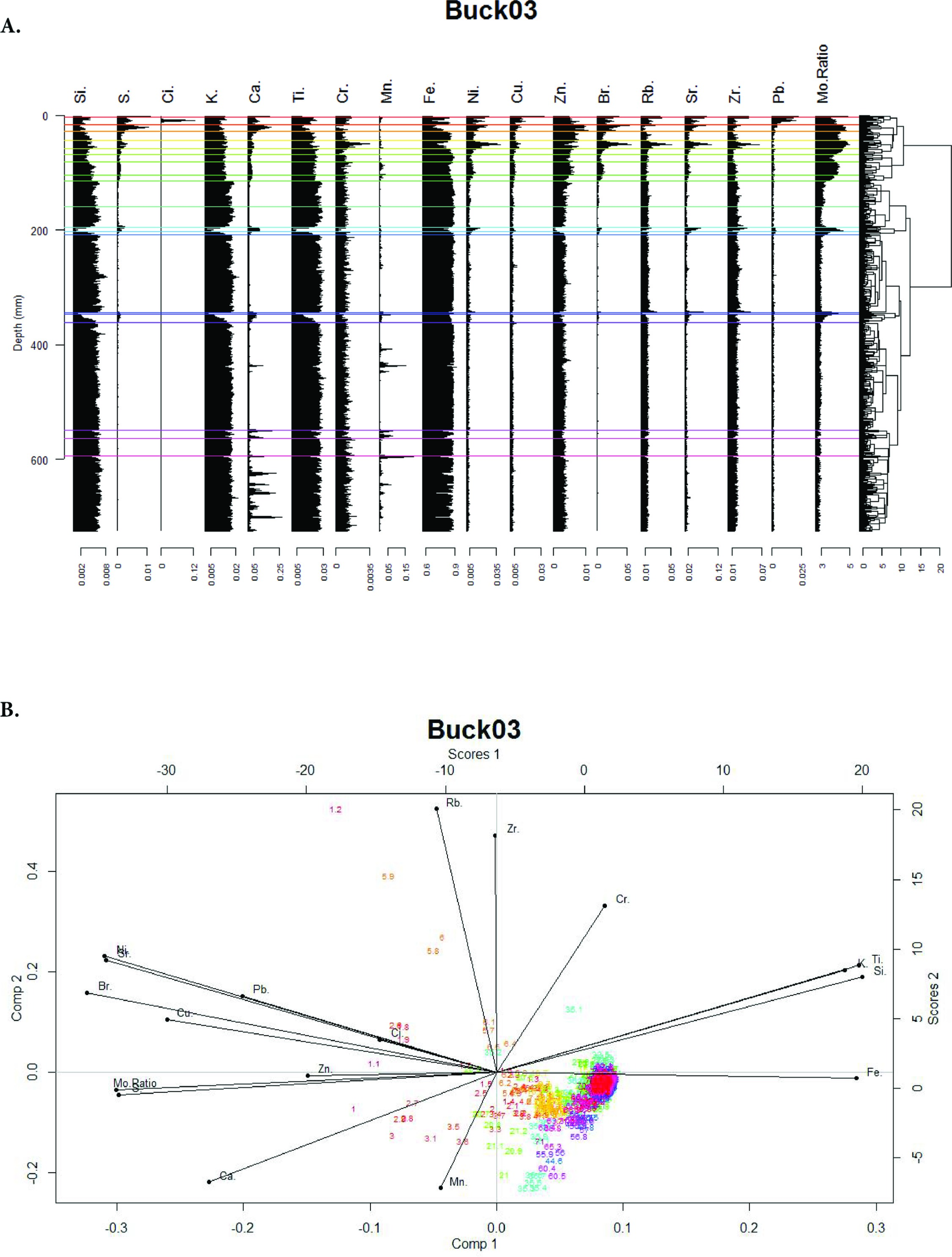

Supplement: S6 Fig — Demonstrates: (A) geochemical elements indicating similarities, and (B) PCA biplot showing concentrations of elements in groups based on geochemical grouping. Coloured numbers refer to the sample depth. (TIF) [file pone.0224011.s008.tif]

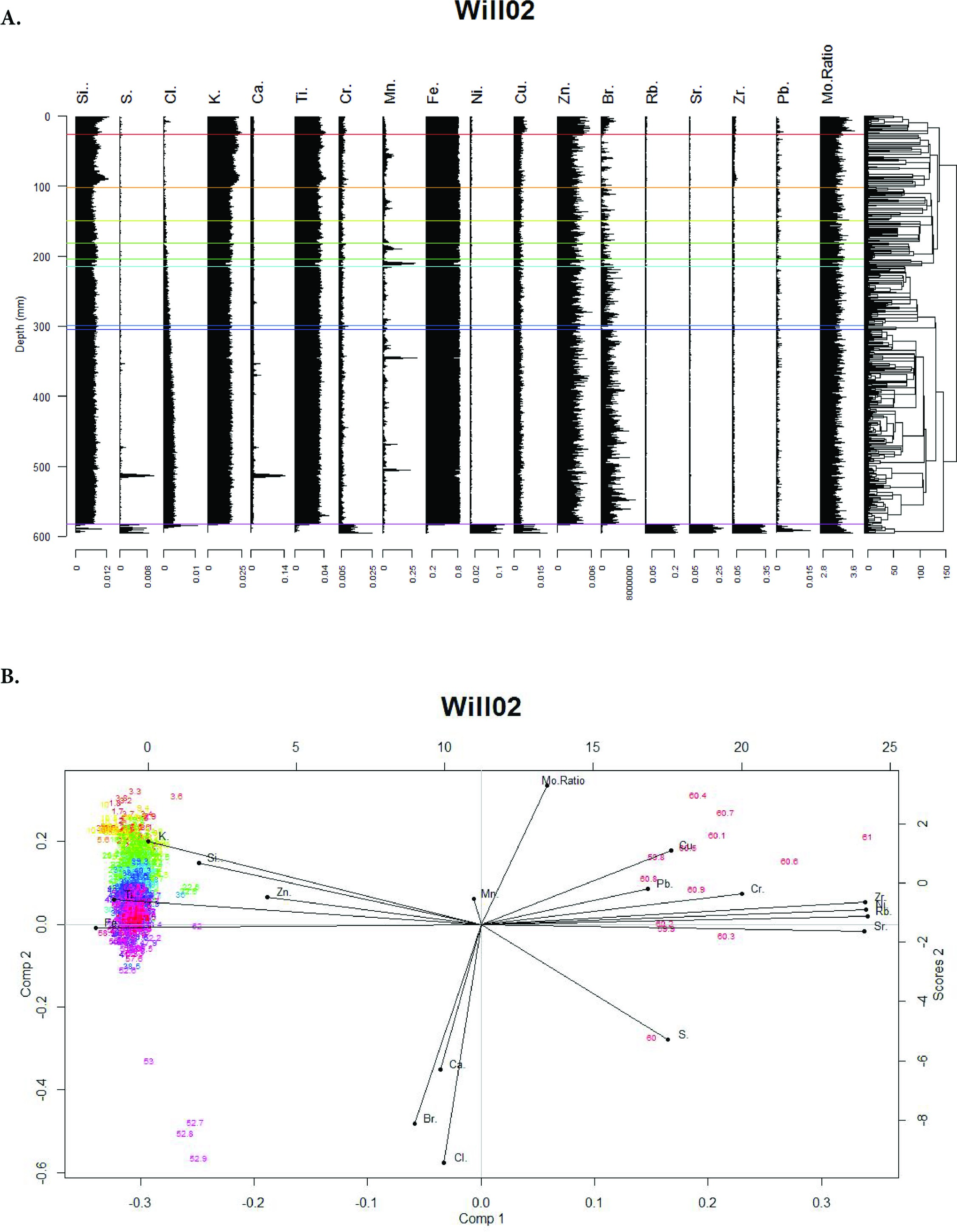

Supplement: S7 Fig — Demonstrates: (A) geochemical elements indicating similarities and (B) PCA biplot showing concentrations of elements in groups based on geochemical grouping. Coloured numbers refer to the sample depth. (TIF) [file pone.0224011.s009.tif]

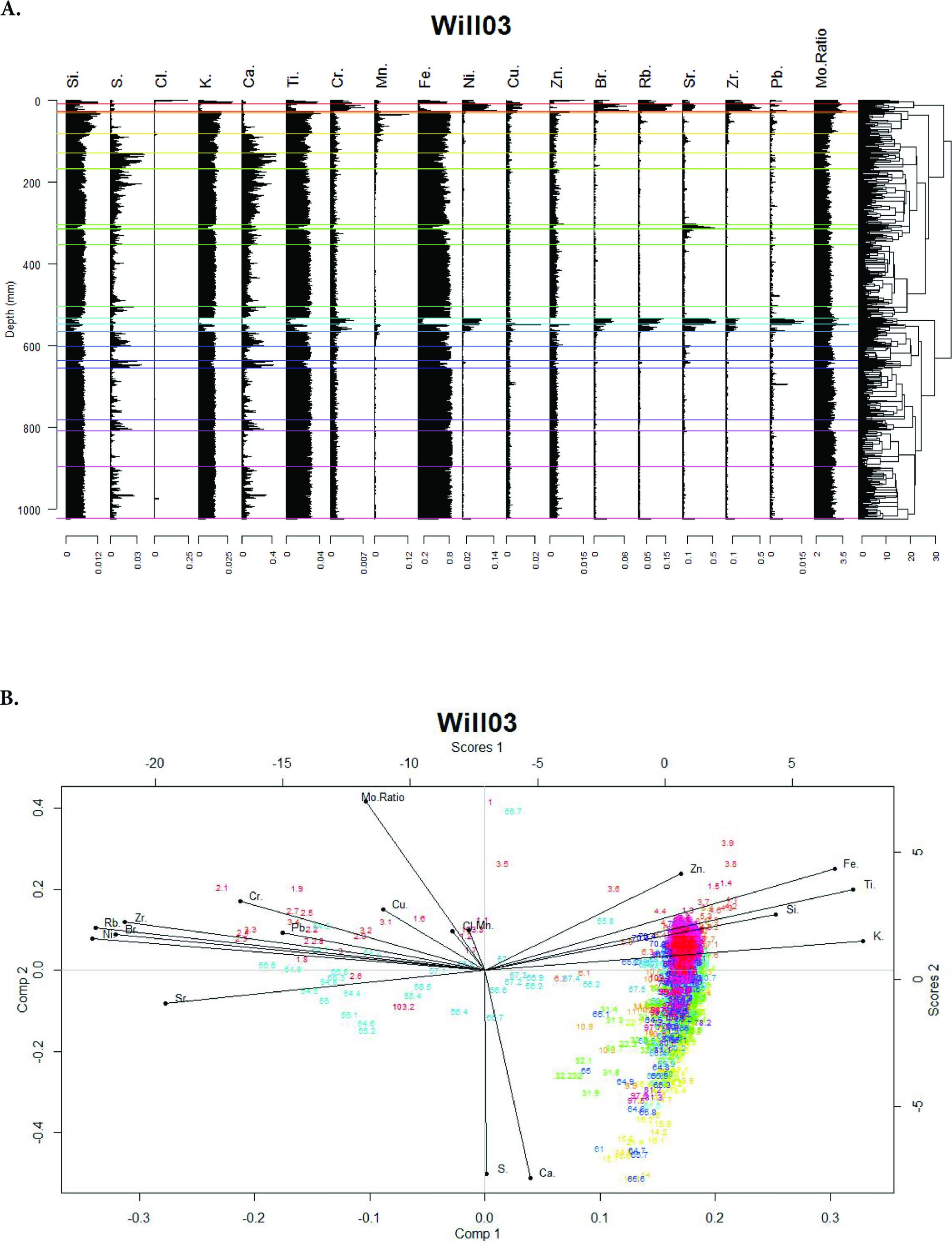

Supplement: S8 Fig — Demonstrates: (A) geochemical elements indicating similarities and (B) PCA biplot showing concentrations of elements in groups based on geochemical grouping. Coloured numbers refer to the sample depth. (TIF) [file pone.0224011.s010.tif]
